# Role of histamine-mediated macrophage differentiation in clearance of metastatic bacterial infection

**DOI:** 10.3389/fimmu.2023.1290191

**Published:** 2023-11-14

**Authors:** Kwang H. Kim, Donghwan Park, Soo Young Cho, Yejin Cho, Buhyun Lee, Haengdueng Jeong, Yura Lee, Yourim Lee, Ki Taek Nam

**Affiliations:** ^1^ Department of Biomedical Sciences, Yonsei University College of Medicine, Seoul, Republic of Korea; ^2^ Department of Molecular and Life Science, Hanyang University College of Science and Convergence Technology, Ansan, Republic of Korea; ^3^ Department of Pathology, Seoul National University Hospital, Seoul, Republic of Korea

**Keywords:** histamine, macrophage differentiation, bacterial infection, single-cell RNA sequencing, peritoneal cells

## Abstract

Macrophages are highly heterogeneous immune cells with a role in maintaining tissue homeostasis, especially in activating the defense response to bacterial infection. Using flow cytometric and single-cell RNA-sequencing analyses of peritoneal cells, we here show that small peritoneal macrophage and immature macrophage populations are enriched in histamine-deficient (*Hdc* ^-/-^) mice, characterized by a CD11b^mi^F4/80^lo^CCR2^+^MHCII^hi^ and CD11b^lo^F4/80^mi^THBS1^+^IL-1α^+^ phenotype, respectively. Molecular characterization revealed that immature macrophages represent an abnormally differentiated form of large peritoneal macrophages with strong inflammatory properties. Furthermore, deficiency in histamine signaling resulted in significant impairment of the phagocytic activity of peritoneal macrophage populations, conferring high susceptibility to bacterial infection. Collectively, this study reveals the importance of histamine signaling in macrophage differentiation at the molecular level to maintain tissue homeostasis, offering a potential therapeutic target for bacterial infection-mediated diseases.

## 1 Introduction

Macrophages are mature, differentiated leukocytes that play essential roles in maintaining tissue homeostasis by clearance of bacteria that penetrate the tissue (1). Macrophages are also crucial factors in tissue repair and development processes (2), thereby playing an important role in the pathology of various diseases such as infection, chronic inflammatory diseases, neurodegenerative disease, autoinflammatory disease, obesity, cardiovascular disease, and cancer (3–5). Recent evidence indicates that macrophages are a highly heterogeneous population that exhibit plasticity in response to various stimuli from adjacent environments. For example, intestinal macrophages defend the intestinal barrier as a prime site for microorganism contact, which is achieved by bacterial sampling in the luminal region, tissue remodeling, and elimination of penetrating bacteria (6). Thus, an accurate understanding of macrophage phenotypes and their functions in different tissues is critical for the development of effective therapeutic approaches.

The peritoneal cavity is a myeloid cell niche that contains various populations of macrophages playing key roles in the inflammatory response (7, 8). Peritoneal macrophages (PMs) play a role in innate and adaptive immunity in the peritoneal cavity, as one of the most common sources of macrophages, along with the bone marrow and spleen. Approximately 30% of peritoneal cells are composed of PMs (9), which are mainly characterized as F4/80^high^MHCII^low^ large peritoneal macrophages (LPMs) and F4/80^low^MHCII^high^ small peritoneal macrophages (SPMs) (10). PMs play an important role in maintaining tissue homeostasis, with a primary role in regulating defense mechanisms against infections such as those caused by bacteria, viruses, fungi, and parasites (11). Phagocytosis of infected PMs is a major mechanism in the clearance of invading pathogens, which is important to prevent peritonitis (12). When peritonitis occurs, bacteria rapidly spread to the blood, inducing sepsis, and finally resulting in death due to multiple organ failure. Studies with models of inflammatory bowel disease demonstrated that PMs also play an important role in gut homeostasis by their direct recruitment to the intestinal wall (13, 14). As PMs are the most abundant population in the peritoneum and are closely related to macrophages that can be directly recruited to other tissues such as the visceral organs (15) and tumors (16), their molecular identification and functional/developmental characterization are important to gain a broader understanding of the diverse functions and regulation mechanisms of macrophages.

Histamine is an organic molecule based on the ammonia (NH_3_) structure, which plays various biological roles such as in the regulation of immune responses (17), neurotransmission (18), and gastric acid secretion (19). In the innate immune system, histamine signaling is a primary factor in the allergic responses by mast cells, and further promotes the differentiation of macrophages (20, 21), monocyte-derived dendritic cells (DCs) (22), hematopoietic stem cells (23), and human M1-type macrophages (24). Endogenous histamine is generated through the decarboxylation of histidine by histidine decarboxylase (HDC) (25). Therefore, HDC is critical for histamine production and macrophage differentiation. Studies in HDC-deficient mice demonstrated the importance of the histamine signal for myeloid cell differentiation, in which HDC deficiency led to the accumulation of immunosuppressive CD11b^+^Ly6G^+^ immature myeloid cells that promote carcinogenesis (26) and inhibited CD8^+^ T cell proliferation in glioma models (27). Although histamine signaling was confirmed to be required for the activation of phagocytosis in studies with histamine receptor-deficient macrophages (28, 29), the molecular mechanism by which histamine signaling induces the complete differentiation of macrophages to achieve their phagocytic function remains poorly understood.

The stomach is in a highly acidic condition due to the secretion of gastric acid by parietal cells (30). Our previous study suggested that histamine signal deficiency results in chronic inflammation of the stomach and hypertrophic gastropathy, and these effects were largely attributed to the consequent loss of the phagocytic activity of stomach-specific macrophages (31). Therefore, we hypothesized that histamine signal deficiency alters the function and phenotype of the PM population. However, this previous study was limited by only focusing on the role of stomach-specific macrophages and the general importance of macrophages in the suppression of histamine signaling has been largely overlooked. Therefore, similar studies are needed in other tissues to determine whether histamine is required for the full differentiation of macrophages.

Here, we reveal the role of histamine in macrophage differentiation and the functional properties of differentiated macrophage populations using single-cell RNA sequencing (scRNA-seq) and flow-cytometric analysis in PMs. Specifically, we identified unique PM populations and changes in phagocytic gene expression in a histamine-deficient (*Hdc* ^-/-^) mouse model. Moreover, we demonstrate that the macrophages of *Hdc* ^-/-^ mice have impaired function and differentiation ability, resulting in an increased susceptibility to bacterial infection. This study thus highlights the importance of histamine-mediated signaling for macrophages to exert their function in suppressing invading bacteria.

## 2 Materials and methods

### 2.1 Mice

The animal experiments were approved by the Institutional Animal Care and Use Committee of Yonsei University (2021–0057) and were compliant with the Guide for the Care and Use of Laboratory Animals. Wild-type FVB/NJ mice were purchased from The Jackson Laboratory (Bar Harbor, ME, USA), and *Hdc* ^-/-^ mice were a kind gift from Dr. Timothy C. Wang (Columbia University, NY, USA). The mice were maintained in a specific pathogen-free facility or biosafety level-2 facility (for the experiments with *Listeria monocytogenes* (*L. monocytogenes*) infection), and maintained under a 12-h light cycle and provided PicoLab Rodent Diet 20 (LabDiet, St. Louis, MO, USA). Four weeks after birth, the offspring of *Hdc* ^-/-^ mice were separated from the mother cage and genotyped with the primers WT_S (GAGCACTGTCAGCGAATCCAC), WT_AS (GGCCGTGAGATAAGC GTGACC), and HDC_AS (TGGGATTAGATAA ATGC CTGCTCT).

### 2.2 Flow cytometry

The total peritoneal cells from mice were isolated using a 10-ml syringe with ice-cold PBS, and cells were blocked with Fc Block (BD) diluted in 100 μL FACS buffer (0.5% FBS, 1 mM EDTA, 0.05% NaN_3_ in PBS) at 2 μL/10^6^ cells for 10 min at 4°C, followed by washing with FACS buffer to remove residual Fc Block. Cell surface staining was performed in FACS buffer containing an antibody cocktail on ice for 1 h. After washing with FACS buffer three times, the cells were subjected to flow cytometry using the BD LSR Fortessa flow cytometer, and data were analyzed using FlowJo software.

For intracellular staining, the Fc-blocked cell surface was stained with an antibody cocktail on ice for 1 h. After washing with FACS buffer, the cells were fixed using Cytofix/Cytoperm solution (BD Biosciences) for 20 min on ice, followed by washing with Perm/Wash solution (BD Biosciences). Intracellular staining was performed using PE-IL-1α (BioLegend) or primary anti-THBS1 (Invitrogen), followed by secondary PE-anti-rabbit-IgG (BioLegend) or PE-isotype control antibody (BioLegend) for 1 h on ice. The cells were washed twice with Perm/Wash solution and analyzed by flow cytometry using the BD LSR Fortessa system.

### 2.3 ScRNA-seq of mouse peritoneal cells

Single mouse peritoneal cells were prepared for scRNA-seq on a LUNA-FL Automated Fluorescence Cell Counter (Logos Biosystems) according to the 10× Genomics Single Cell Protocols Cell Preparation Guide and Guidelines for Optimal Sample Preparation Flowchart (Documents CG00053 and CG000126, respectively). To obtain the clearest results, we performed flow cytometry on the peritoneal cells. Based on these results, the 10× Single Cell 3′ v3 scRNA-seq protocol was performed with cells isolated from one male *Hdc* ^+/+^ mouse and one male *Hdc* ^-/-^ mouse; 5375 and 5760 cells, respectively, were initially profiled in each group, with a median of approximately 15,000 genes detected per cell. After removing low-quality cells (mitochondrial gene ratio > 0.1 or <500 genes expressed), 4704 *Hdc* ^+/+^ cells and 5086 *Hdc* ^-/-^ cells were subjected to quality control for further analysis. Libraries were prepared using the chromium controller according to the 10× Single Cell 3′ v3 protocol (10x Genomics, Pleasanton, CA, USA). In brief, cell suspensions were diluted in nuclease-free water to achieve a targeted cell count of 10,000. The cell suspension was mixed with the master mix, loaded with single-cell 3′ gel beads, and partitioned into a single-cell 3′ chip. RNA transcripts from single cells were barcoded and reverse-transcribed within the droplets. cDNA molecules were pooled, subjected to an end-repair process, followed by the addition of a single ‘A’ base, and then the adapters were ligated. The products were purified and enriched using PCR to create the final cDNA library. The purified libraries were quantified using quantitative PCR according to the qPCR Quantification Protocol Guide (KAPA) and qualified using an Agilent Technologies 4200 TapeStation. Finally, the libraries were sequenced using the HiSeq platform (Illumina, San Diego, CA, USA), with a read length of 28 bp for read 1 (cell barcode and unique molecular identifiers [UMIs]), 8 bp for the index read (sample barcode), and 91 bp for read 2 (RNA read).

The scRNA-seq data were processed using Cellranger (release 3). The reads were mapped to the mm10 reference genome, supplied by 10× Genomics. A gene count matrix was generated from UMIs, and functions in the Seurat package were used to remove low-quality cells and normalize the scRNA-seq data (32) according to the following criteria: 1) number of expressed genes <500 or >5000, and 2) >10% UMIs mapped to mitochondrial genes. The data were then normalized using the “normalizeData” function with the “LogNormalize” setting. Finally, we identified 4704 cells and 15,409 genes in the knockout condition (*Hdc* ^-/-^), and 5086 cells and 15,220 genes in the wild-type condition (*Hdc* ^+/+^).

We performed principal component analysis to select variable genes to reduce the dimensions of the data and to construct a two-dimensional representation using UMAP. We then utilized the “FindClusters” function in the Seurat packages to conduct the cell clustering analysis through embedding cells into a graph structure in the principal components space. Finally, we identified 16 clusters from the *Hdc* ^+/+^ and *Hdc* ^-/-^ datasets. To identify DEGs associated with each cluster, the standard AUC classifier was used with the “FindAllMarkers” function, and genes with an AUC ≥ 0.85 were selected. All clusters were annotated according to the immune cell type within each of the DEGs based on the expression of known immune cell marker genes that were manually curated. Finally, we annotated the 11 immune cell types. HALLMARK database analysis were conducted from Molecular Signatures Database (MSigDB) (33).

Trajectory analysis was performed to track the cell transition status. The cells were re-processed to remove genes with a low UMI count and re-normalized to the library size. Dimensionality reduction and trajectory construction were performed using the R package Monocle (34) with default parameters. The cells were placed onto a pseudo-time trajectory using the “orderCell” function.

### 2.4 Phagocytosis assay

The PMs were suspended in RPMI-1640 medium (5% FBS) at a density of 2 × 10^5^ cells/mL. Each group of cells was seeded into 12-well plates and incubated for 6 h in a 37°C, 5% CO_2_ incubator. A total of 1 × 10^6^ FluoSpheres polystyrene beads (1.0 μm; 580/605, Invitrogen) were added to each well and incubated with the cells for 30 min with gentle shaking. For flow cytometric analysis, the harvested cells were washed with FACS buffer and blocked with Fc Block (BD) for 10 min at 4°C. Cell surface staining was performed in FACS buffer containing FITC-F4/80 on ice for 1 h. After washing with FACS buffer three times, the cells were analyzed using a BD FACSverse cytometer. FluoSpheres polystyrene beads were detected according to PE fluorescence.

To investigate the *in vivo* phagocytic acitivity of peritoneal macrophages in *Hdc* ^-/-^ mice, we conducted fluorescence bead phagocytosis assay with modification (35). For the assay, 4-week-old *Hdc* ^+/+^ and *Hdc* ^-/-^ mice were intraperitoneally injected with 1 × 10^7^ of 1.0-μm FluoSpheres polystyrene beads (580/605)/200 μL and left to react for 30 or 60 min. All mice were then sacrificed and FACS analysis was performed as described above.

### 2.5 Phagocytosis of *L. monocytogenes*

*L. monocytogenes* (strain 10403S, American Type Culture Collection) were cultured in brain heart infusion broth (BD, 237500), and cells were labeled with 1 μM of CFSE (eBioscience, 65-0850-84) for 20 min at 25°C in the dark. CFSE-labeled bacteria were washed with PBS, centrifuged at 1700 ×*g* for 10 min, and incubated with 100% FBS for 10 min at 37°C. CFSE-labeled *L. monocytogenes* were washed again, added to each PM-seeded well at 1 × 10^8^ CFU, and incubated for 30 min in a 37°C, 5% CO_2_ incubator. The subsequent steps were the same as those described above for the flow cytometric analysis.

### 2.6 H&E and immunohistochemical staining of the liver

For H&E staining, the mouse liver tissues were fixed with ice-cold 4% paraformaldehyde and then embedded in paraffin. The tissues were sectioned at 5 μm thickness; deparaffinized with xylene three times for 20 min each, 100% ethanol three times for 10 min each, 90% ethanol twice for 10 min each, and 75% ethanol for 10 min; and then stained with H&E. The stained liver tissue slides were dehydrated and mounted with Shandon Synthetic Mount (Thermo, 6769007) for observation.

For immunohistochemistry, paraffin-embedded specimens were sectioned at 3 μm, deparaffinized, and hydrated in PBS. Antigens were then retrieved for 15 min under high pressure in Target Retrieval solution (Dako, S1699). Subsequently, the specimens were chilled on ice for 1 h, washed with PBS three times for 5 min each, and blocked with 3% H_2_O_2_ in PBS for 30 min to quench the endogenous peroxidase. The slides were washed again with PBS, blocked for 2 h at room temperature with Serum-Free Protein Block (Dako, X0909), and probed at 4°C overnight with the anti-neutrophil antibody (Abcam, ab2557) (1/1,000 dilution). Horseradish peroxidase-conjugated anti-rat IgG secondary antibody staining was performed using VECTASTAIN ABC HRP Kit (VECTA labs, PK-4004), and developed with Liquid DAB+ Substrate Chromogen System (Dako, K3468). Finally, the specimens were counterstained with Mayer’s hematoxylin (Dako, S3309) and mounted with Shandon Synthetic Mount for observation.

### 2.7 Quantification and statistical analysis

Statistical analyses were performed using Prism software (GraphPad). To evaluate statistical significance, differences between groups were compared using Student’s t-test. Significance was judged at *P* < 0.05. Statistical details, including the sample size, for each experiment are provided in the relevant figure legends.

## 3 Results

### 3.1 Generation of abnormally differentiated PMs in *Hdc* ^-/-^ mice

**Figure 1:**
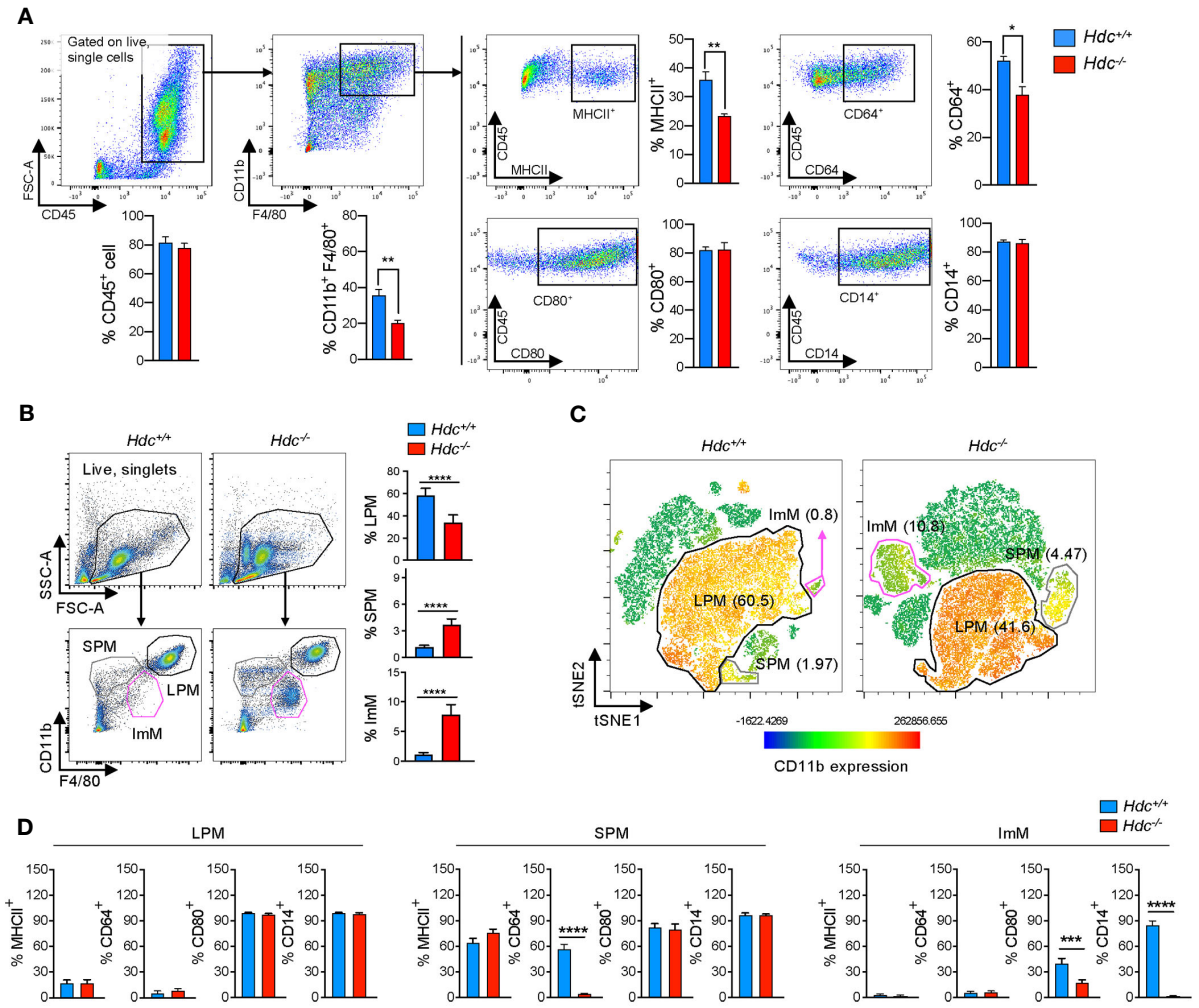
Alteration of macrophage phenotype and a new immature macrophage population identified in *Hdc* ^-/-^ mice. **(A)** Flow cytometric gating strategy to characterize bone marrow-derived macrophages (BMDMs). GM-CSF–treated BMDMs obtained from 4-week-old *Hdc* ^+/+^ and *Hdc* ^-/-^ mice were first gated on live (DAPI^-^), single, and CD45^+^ cells. CD45^+^ cells were further distinguished by CD11b^+^F4/80^+^, and CD80^+^, CD64^+^, CD14^+^, and MHCII^+^ cells were respectively counted (n = 3 mice). **(B)** Flow cytometric gating strategy to characterize mouse peritoneal macrophages (PMs). *Hdc* ^+/+^ and *Hdc* ^-/-^ mouse peritoneal cells were first gated on live (DAPI^-^), single, and CD45^+^ cells, followed by three overlapping gates defined by the relative expression of CD11b and F4/80 to define large peritoneal macrophages (LPMs: CD45^+^CD11b^high^F4/80^high^), small peritoneal macrophages (SPMs: CD45^+^CD11b^mid^F4/80^low^), and immature macrophages (ImMs: CD45^+^CD11b^low^F4/80^mid^), and for the quantification of LPM, SPM, and ImM populations from *Hdc* ^+/+^ and *Hdc* ^-/-^ mouse peritoneal cells (n = 4 mice). **(C)** The t-distributed stochastic neighbor embedding (tSNE) projection from *Hdc* ^+/+^ and *Hdc* ^-/-^ mouse peritoneal cells. LPM, SPM, and ImM populations are highlighted. **(D)** Gated LPM, SPM, and ImM populations further identified by the expression of MHCII, CD64, CD80, and CD14. Results are presented as the mean ± SD; *P < 0.05, **P < 0.01, ***P < 0.001, ****P < 0.0001.

In line with a previous report showing that histamine signal deficiency suppressed myeloid maturation (26), we found that the GM-CSF–mediated differentiation of CD11b^+^F4/80^+^ bone marrow-derived macrophages (BMDMs) was impaired in *Hdc* ^-/-^ mice, and the expression of MHCII and CD64 was also downregulated in *Hdc* ^-/-^ BMDMs compared with that of wild-type (*Hdc* ^+/+^) mice, whereas CD80 and CD14 expression was not affected by histamine deficiency (**Figure 1A**). Flow cytometry analysis showed that histamine deficiency clearly distinguished PMs with various differentiation status: the proportion of LPMs (CD45^+^CD11b^high^F4/80^high^) was decreased, whereas the proportion of SPMs (CD45^+^CD11b^mid^F4/80^low^) was increased in 4-week-old *Hdc* ^-/-^ mice (**Figure 1B**). Notably, an abnormally differentiated immature macrophage (ImM) population, characterized by the CD45^+^CD11b^low^F4/80 ^mid^ phenotype, was enriched in *Hdc* ^-/-^ mice (**Figures 1B, C**). We also found that histamine-mediated macrophage differentiation is required for the expression of CD64 in SPMs and CD80 and CD14 in ImMs (**Figure 1D**). Collectively, these data demonstrate that histamine signaling is important for the complete differentiation of macrophages.

### 3.2 ScRNA profiling of PMs under histamine deficiency

**Figure 2:**
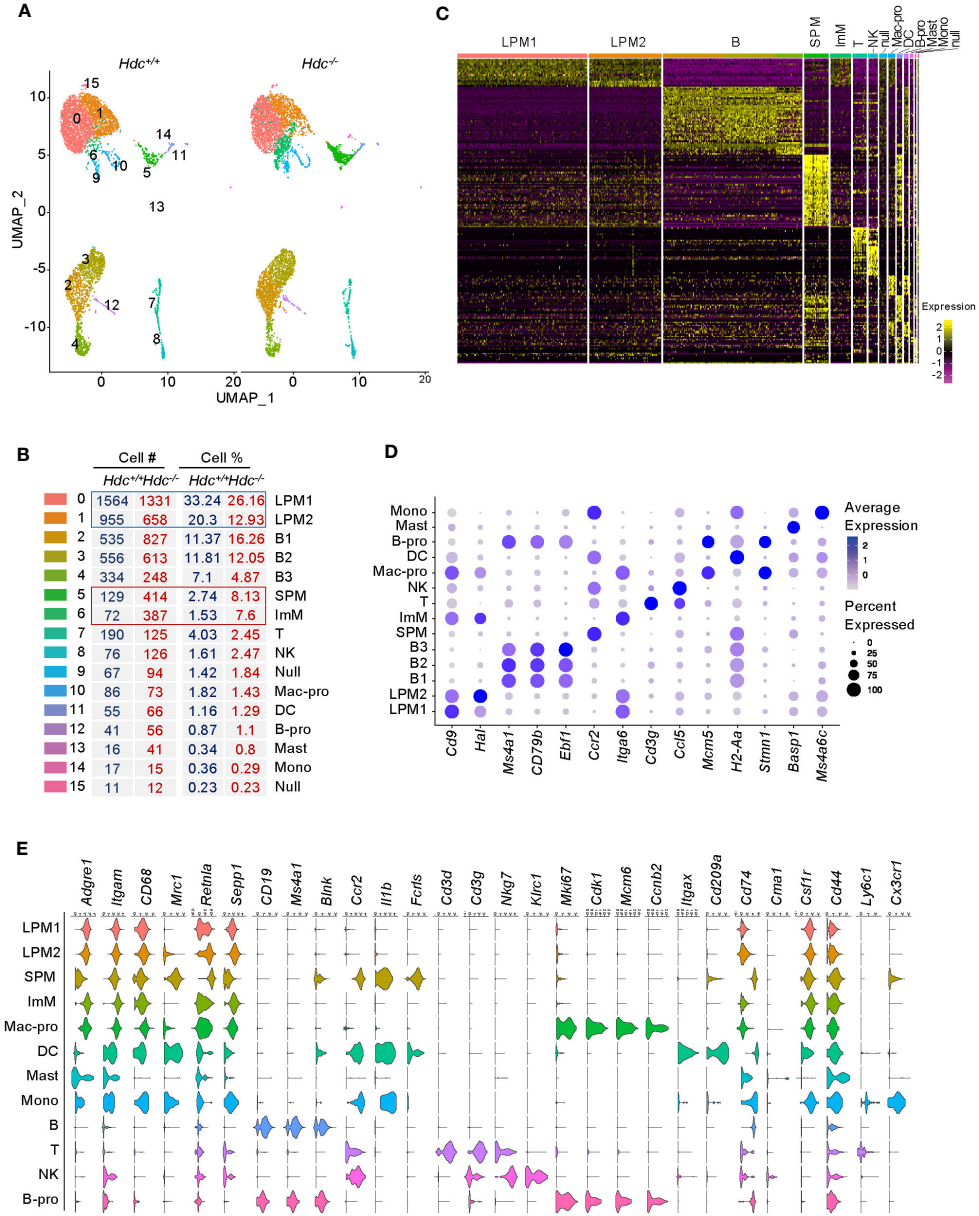
Single-cell transcriptome atlas of mouse peritoneal cells. ScRNA-seq was applied to total peritoneal cells isolated from 4-week-old *Hdc* ^+/+^ and *Hdc* ^-/-^ mice (one mouse per group). **(A)** Uniform manifold approximation and projection (UMAP) plot from 4-week-old *Hdc* ^+/+^ and *Hdc* ^-/-^ mouse peritoneal cells. **(B)** Number and percentage of assigned cell types. LPM1 and 2, large peritoneal macrophages 1 and 2; B 1-3, B cells 1–3; SPM, small peritoneal macrophages; ImM, immature macrophages; T, T cells; NK, natural killer cells; Mac-pro, proliferating macrophages; DC, dendritic cells; B-pro, proliferating B cells; Mast, mast cells; Mono, monocytes; Null, uncharacterized cells. **(C)** Heat map of differentially expressed genes (DEGs) for each cluster in **(A)** with an area under the curve (AUC) cut-off ≥ 0.85. **(D)** Average expression levels (represented by the color scheme) and percentage of cells (represented by the spot size) of the top-expressing genes across the 14 main clusters (excluding the Null cluster). **(E)** Violin plots of the expression levels of representative marker genes. The *y-*axis shows the log scale–normalized read count. Macrophage markers = *Adgre1*, *Itgam*, *CD68*, *Mrc1*, *Retnla*, *Sepp*; B cell markers = *CD19*, *Ms4a1*, *Blnk*; inflammation markers = *Ccr2*, *Il1b*; T cell markers = *Cd3d*, *Cd3g*; NK cell markers = *Nkg7*, *Klrc1*; proliferation markers = *Mki67*, *Cdk1*, *Mcm6*, *Ccnb2*; DC markers = *Itgax*, *Cd209a*; antigen presentation markers = *Cd74*; mast cell marker = *Cma1*; monocyte markers = *Csf1r*, *Cd44*, *Ly6c1*, *Cx3cr1*.

To further characterize the alteration of the macrophage phenotype at the molecular level in a histamine-deficient condition, we applied scRNA-seq to total peritoneal cells isolated from 4-week-old *Hdc* ^+/+^ and *Hdc* ^-/-^ mice (one mouse per group). To identify cell populations based on differentially expressed gene (DEG) patterns, we performed unsupervised cell clustering using the Seurat software suite. Uniform manifold approximation and projection (UMAP) identified a total of 16 cell clusters (**Figure 2A**), representing B cells (B, clusters 2–4), T cells (T, cluster 7), natural killer cells (cluster 8), proliferating macrophages (Mac-pro, cluster 10), DCs (cluster 11), proliferating B cells (B-pro, cluster 12), mast cells (Mast, cluster 13), monocytes (mono, cluster 14), undistinguishable clusters (Null, clusters 9 and 15) (**Figure 2B**), and four macrophage clusters (clusters 0, 1, 5, and 6) based on marker expression (**Supplementary Table S1**). The neutrophil cluster was not identified under this experimental condition, in line with a previous report (36). Clusters 0 and 1 comprised the major population, which we named LPM1 and LPM2, respectively, and were reduced in the *Hdc* ^-/-^ *mouse* compared with the *Hdc* ^+/+^ mouse. Conversely, clusters 5 and 6 were enriched in *Hdc* ^-/-^ mouse and were characterized as SPM and ImM, respectively (**Figure 2B**). In total, 259 DEGs best characterized the cells in these clusters, with an area under the curve (AUC) value ≥ 0.85 (**Figure 2C**; **Supplementary Table S2**). The LPM1 and LPM2 populations showed similar gene expression patterns, although the expression of M2 macrophage-like marker genes (*Cbr2, Folr2*, and *Sepp1*) was upregulated in the LPM2 population. The ImM population showed a gene expression pattern similar to that of LPM1 and LPM2, but with lower overall gene expression levels. Although the SPM cluster was characterized as a macrophage population, the gene expression patterns were more similar to those of the DC and mono clusters (**Figure 2C**). Among the top expressed genes in each cluster, *Cd9* was more highly expressed in LPM1 but was also expressed in other macrophage clusters. Although histidine ammonia-lyase (*Hal*) is not a typical macrophage marker, this gene was highly expressed in the LPM2, LPM1, ImM, and Mac-pro clusters. Membrane spanning 4-domains A1 (*Ms4a1)*, *Cd79b*, and early B-cell factor 1 (*Ebf1)* were highly expressed in B cell clusters. *Ccr2* and *Itga6* were the most highly expressed genes in the SPM and ImM clusters, respectively. Proliferating cell clusters (Mac-pro and B-pro) highly expressed *Mcm5* and *Stmn1*, and the DC cluster highly expressed the antigen presentation-related gene *H2-Aa* (**Figure 2D**).

**Figure 3:**
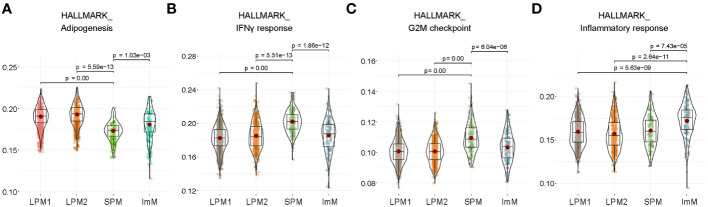
Functional properties of large peritoneal macrophages (LPMs), small peritoneal macrophages (SPMs), and immature macrophages (ImMs). **(A–D)** HALLMARK analysis of adipogenesis **(A)**, IFN-γ response **(B)**, G2M checkpoint **(C)**, and inflammatory response **(D)** pathways of LPM1, LPM2, SPM, and ImM. Each cluster is visualized by a violin plot.

To further distinguish SPM and ImM from the other clusters, we used well-known phenotypic and functional markers for macrophages, B cells, T cells, natural killer cells, proliferating cells, DCs, antigen-presenting cells, mast cells, and monocytes to identify DEGs between groups (**Figure 2E**). Clusters LPM1 and LPM2 were predominant, with high expression of the macrophage markers *Adgre1* (F4/80) and *Itgam* (CD11b), but low expression of the antigen-presenting cell marker *Cd74* (MHCII). Therefore, LPM1 and LPM2 were matched with the CD11b^high^F4/80^high^MHCII^low^ (LPM) population obtained using flow cytometry (**Figures 1B, D**). The expression level of *Adgre1* in SPM was lower than that of the other macrophage clusters, whereas the expression level of *Cd74* was relatively high in the SPM cluster (**Figure 2E**), which is consistent with the expression levels of these genes found in the CD11b^mid^F4/80^low^MHCII^high^ (SPM) population by flow cytometry (**Figures 1B, D**). Although the heat map of DEGs identified ImM as a separate cluster (**Figure 2C**), the expression patterns of macrophage marker genes in this cluster were comparable to those of LPM1 and LPM2 (**Figure 2E**). HALLMARK database analysis demonstrated the upregulation of adipogenesis, glycolysis, heme metabolism, angiogenesis, and Notch signaling pathways in LPM clusters compared to the SPM cluster. This indicated that LPMs are mainly composed of tissue-resident macrophages that maintain tissue homeostasis (**Figure 3A**; **Supplementary Figure 1A**). In the SPM cluster, the complement system was upregulated, whereas the ROS pathway, coagulation, immune response, and cell cycle pathways were downregulated, including the IFN-γ response, IFN-α response, allograft rejection, IL-2–STAT5 signaling, and G2M checkpoint (**Figures 3B, C**; **Supplementary Figures 1B, C**). The overall signaling pathways enriched in the ImM cluster were similar to those enriched in the LPM clusters. However, pathways related to the inflammatory response were the most significantly upregulated in the ImM cluster compared to other clusters in the naive condition (**Figure 3D**), indicating that ImMs potentially induce inflammation despite the presence of tissue-resident macrophages.

### 3.3 Characterization of the SPM and ImM clusters

**Figure 4:**
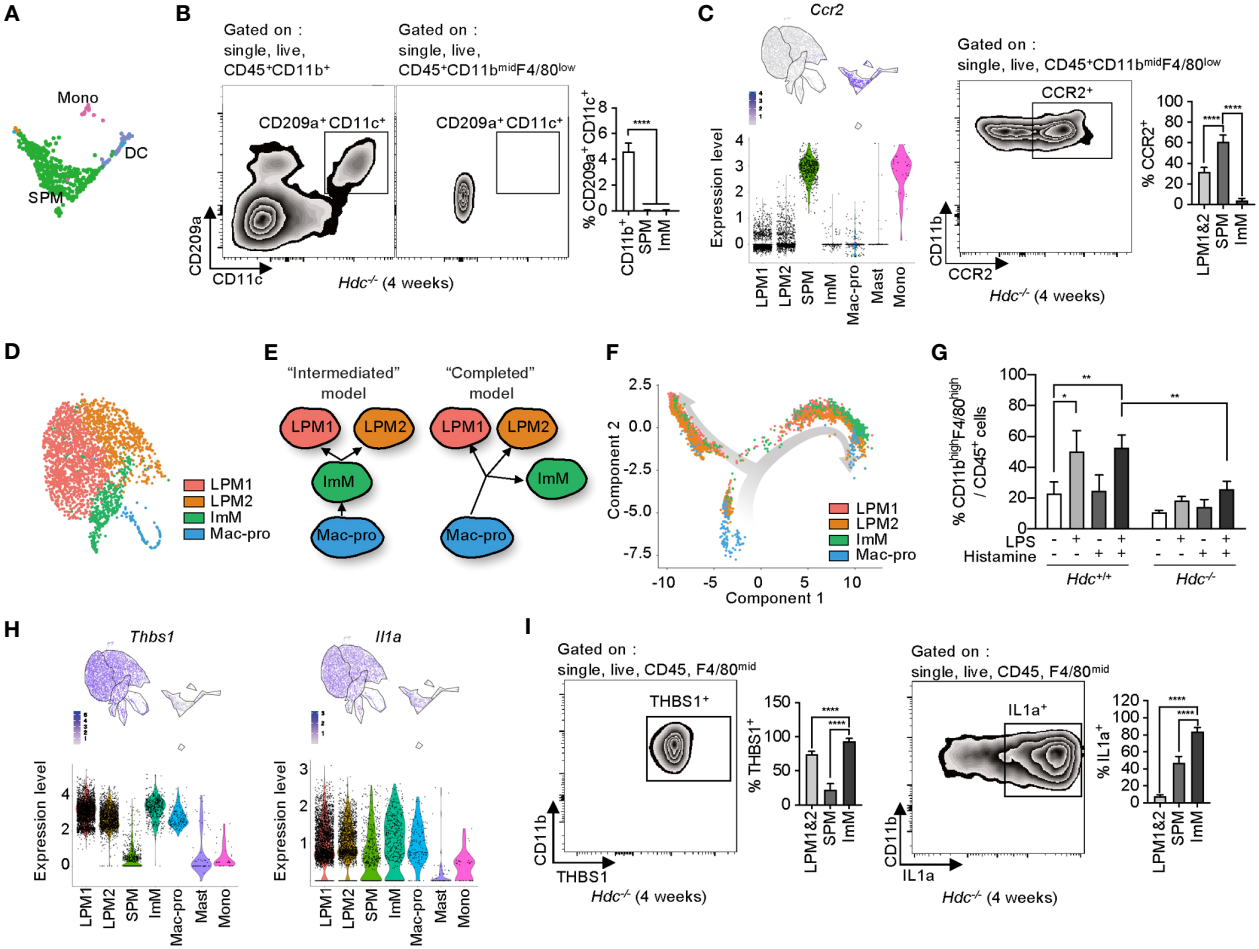
Phenotypic identification of small peritoneal macrophages (SPMs) and immature macrophages (ImMs). **(A)** Highlighted SPM, monocyte (Mono), and dendritic cell **(DC)** clusters from **Figure 2A**. **(B)** DCs were gated by single, live, CD45, CD11b, CD209a, and CD11c cells of 4-week-old *Hdc* ^-/-^ mouse peritoneal macrophages. DCs were distinguished from the CD11b^mid^F4/80^low^ population. Left and middle: representative zebra plot. Right: bar graph of the CD209a^+^CD11c^+^ cell percentages in each population (n = 5 mice). **(C)** Expression level of *Ccr2* on uniform manifold approximation and projection (UMAP) and violin plots (left), representative flow cytometric identification of CCR2^+^ cells from the CD11b^mid^F4/80^low^ population (middle), and quantification of CCR2^+^ large peritoneal macrophage (LPM), SPM, and ImM populations (n = 5 mice). **(D)** Highlighted LPM1, LPM2, ImM, and Mac-pro clusters from **Figure 2A**. **(E)** Schematic of two possible models for the origin of ImMs. **(F)** Unsupervised trajectory ordering single cells used for LPM1, LPM2, ImM, and Mac-pro. **(G)** Peritoneal cells from 4-week-old *Hdc* ^+/+^ and *Hdc* ^-/-^ mice treated with 100 ng/mL of LPS and/or 10^-6^ M of histamine for 2 days *in vitro*. The percentage of CD45^+^CD11b^high^F4/80^high^ cells was calculated (n = 4 mice). **(H)** Expression levels of *Thbs1* and *Il1a* on UMAP and violin plots. **(I)** Protein expression of THBS1 and IL-1α validated using flow cytometry in LPM1, LPM2, SPM, and ImM populations from 4-week-old *Hdc* ^-/-^ mouse peritoneal cells (n = 5 mice). Results are presented as the mean ± SD. *P < 0.05, **P < 0.01, ****P < 0.0001.

The SPM cluster was characterized as macrophages, but was separated from other macrophage clusters and was closer to the DC and Mono clusters according to gene expression patterns (**Figure 4A**), despite the lack of expression of DC marker genes such as *Cd209a*, *Itgax*, and *Mgl2* (**Supplementary Figure 2A**). Flow cytometry of total peritoneal cells from 4-week-old *Hdc* ^-/-^ mice showed that CD209a^+^CD11c^+^ DCs accounted for 4% of total CD11b^+^ cells, whereas there was no DC population identified in the CD11b^mid^F4/80^low^ SPM population (**Figure 4B**). The SPM cluster highly expressed *Hopx* (a regulator of primitive hematopoiesis) (37), along with the pro-inflammatory cytokines *Tnf* and *Il6*, but showed minimal expression of *Vsig4*, which participates in the phagocytosis of bacteria (**Supplementary Figure 2B**) (38), and *Socs3* (**Figure S2C**). *Ccr2*, which is putatively expressed on classical monocytes (39), was also highly expressed in the SPM cluster, and approximately 60% of CD11b^mid^F4/80^low^ SPM cells expressed CCR2 in the peritoneal cells of 4-week-old *Hdc* ^-/-^ mice (**Figure 4C**).

The LPM1, LPM2, ImM, and Mac-pro clusters were closely associated in the UMAP space (**Figure 4D**) and showed similar DEG profiles (**Figure 2C**). In view of the low-level expression of macrophage marker genes in ImM compared to that of the LPM1 and LPM2 clusters, we hypothesized two possibilities: 1) the intermediate form model, in which ImM is an intermediate cell type between Mac-pro and ImM with the ability to differentiate into LPM1 and LPM2; and 2) the completed form model, in which Mac-pro directly differentiates into LPM1, LPM2, and ImM, so that ImM is not an intermediate form but rather represents a population that is immobilized as immature macrophages (**Figure 4E**). To distinguish between these models, we performed a trajectory analysis using the Monocle toolkit with pseudo-time reconstitution of the LPM1, LPM2, ImM, and Mac-pro populations. The majority of ImM cells were located at the endpoint of the trajectory rather than between the Mac-pro and LPM1 and LPM2 clusters (**Figure 4F**), suggesting that ImM may be a terminally differentiated immature form of LPMs. To validate this possibility, total peritoneal cells of 4-week-old *Hdc* ^+/+^ and *Hdc* ^-/-^ mice were stimulated with LPS and/or histamine for 2 days *in vitro*, and the percentage of CD11b^high^F4/80^high^ cells was determined using flow cytometry. Although LPS/histamine double stimulation partially recovered differentiation in *Hdc* ^-/-^ macrophages, this recovery was far lower than that observed in *Hdc* ^+/+^ macrophages (**Figure 4G**). This suggested that the ImM population represents a terminally differentiated form of macrophages with immature properties.

The ImM cluster more highly expressed thrombospondin-1 (*Thbs1*), which is reported as an LPM-specific gene (40), and *Il1a* compared to the expression levels of these genes in the other macrophage clusters (**Figure 4H**). Consistently, flow cytometry of PMs showed the highest levels of Thbs1 and IL-1α proteins in CD11b^low^F4/80^mid^ ImMs compared to those of other macrophage populations (**Figure 4I**).

### 3.4 Histamine-mediated macrophage differentiation is important for phagocytic function

**Figure 5:**
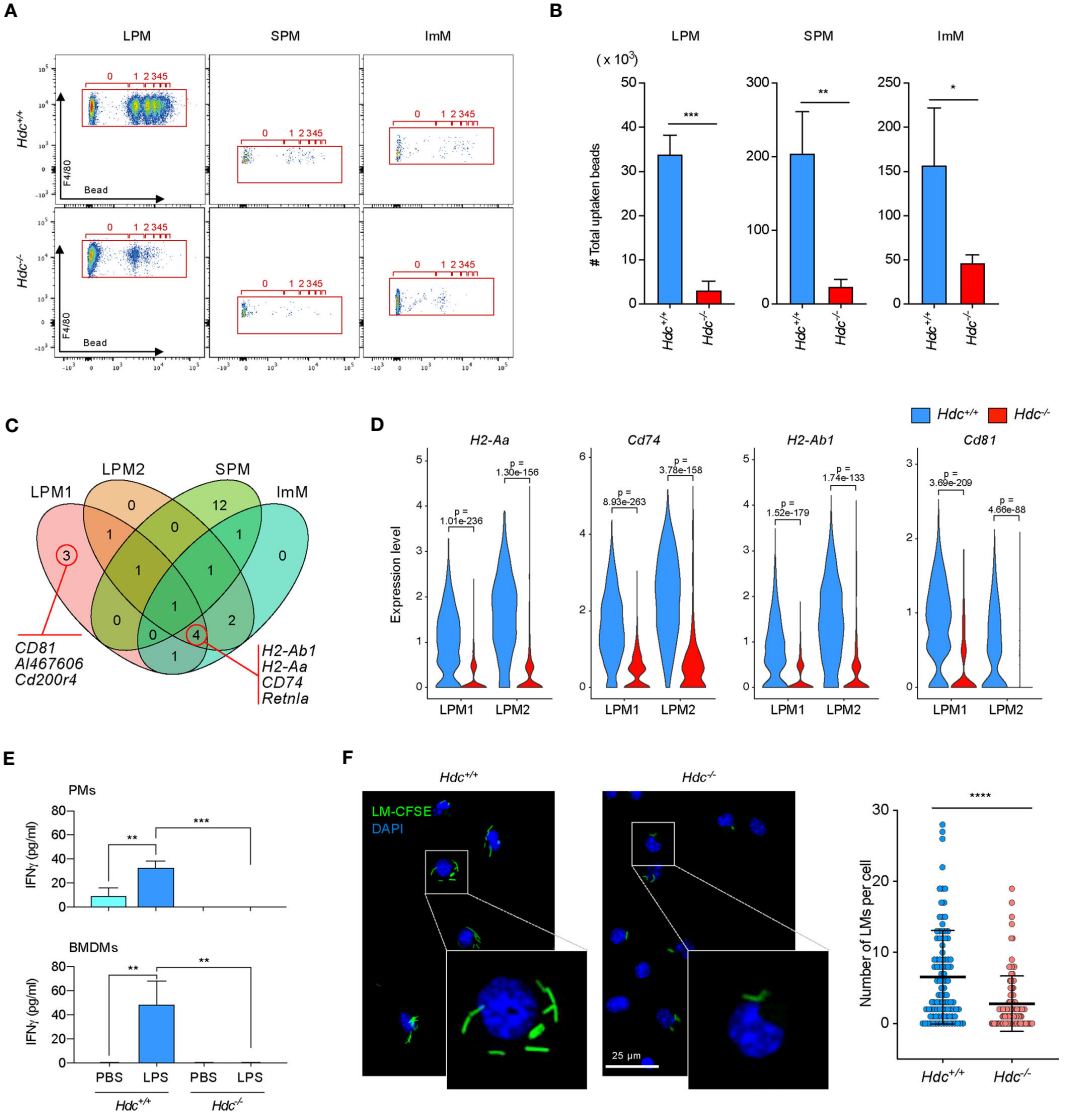
The histamine signal is important for the phagocytic activity of macrophages *in vitro*. **(A, B)** Phagocytosis assay of *Hdc* ^+/+^ and *Hdc* ^-/-^ peritoneal macrophages (PMs) using polystyrene beads. Flow cytometry was performed by gating on live (DAPI^-^), single, F4/80^+^, and beads^+^ cells. The number of beads taken up per large peritoneal macrophage (LPM), small peritoneal macrophage (SPM), and immature macrophage (ImM) is highlighted (red box) **(A)** and the total number of beads taken up is calculated **(B)** (n = 3 mice). **(C)** Selected upregulated genes in *Hdc* ^+/+^ compared with *Hdc* ^-/-^ mice among total differentially expressed genes (DEGs), and their distribution and overlap among the four clusters (LPM1, LPM2, SPM, and ImM) are shown in a Venn diagram. **(D)** Violin plot comparing the expression levels of *H2-Aa, Cd74, H2-Ab1*, and *Cd81* in the LPM1 and LPM2 clusters of *Hdc* ^+/+^ and *Hdc* ^-/-^ mice. **(E)** Cytometric bead array-based measurement of IFN-γ. Four-week-old PMs from *Hdc* ^+/+^ and *Hdc* ^-/-^ mice (top graph) or GM-SCF–treated bone marrow-derived macrophages (BMDMs; bottom graph) were treated with PBS (control) or LPS. The levels of cytokines in each group in the culture supernatant were determined (n = 4 mice). **(F)** *In vitro* phagocytosis assay of *Hdc* ^+/+^ and *Hdc* ^-/-^ PMs using CFSE-labeled *L. monocytogenes* (LM). LM cells containing PMs were visualized by fluorescence microscopy and the number of LMs in each PM was quantified. Results are presented as the mean ± SD. *P < 0.05, **P < 0.01, ***P < 0.001, ****P < 0.0001.

Previous reports showed that histamine is important for the function of various types of macrophages via interacting with histamine receptors. Therefore, we next performed a microbead-based phagocytosis assay in PMs of 4-week-old and 1-year-old *Hdc* ^+/+^ and *Hdc* ^-/-^ mice. To define phagocytic activity in each population, PMs were first gated on CD45^+^CD11b^high^F4/80^high^ (consistent with LPM1 and LPM2), CD45^+^CD11b^low^F4/80^mid^ (consistent with ImM), and CD45^+^CD11b^mid^F4/80^low^ (consistent with SPM), and each population was further examined for F4/80 staining and red-stained microbeads. We detected dramatically impaired phagocytic activity in *Hdc* ^-/-^ LPMs compared with that of *Hdc* ^+/+^ cells. Although the SPM and ImM populations were small, uptake of 1–5 beads per cell was detected in *Hdc* ^+/+^ PMs. Despite enrichment of the SPM and ImM populations in *Hdc* ^-/-^ PMs, most of these cells could not uptake the beads (**Figures 5A, B**).

To identify the histamine signal-mediated genes involved in phagocytosis, we selected upregulated DEGs in *Hdc* ^+/+^ mice relative to those in *Hdc* ^-/-^ mice in each cluster, which were compared with Venn diagram analysis. Phagocytosis and antigen presentation-related genes such as *H2-Ab1, H2-Aa, Cd74* (41), and *Cd81* (42) were upregulated in LPM clusters (**Figure 5C**; **Supplementary Table S3**). Violin plot analysis also confirmed that the expression of phagocytosis-related genes was significantly impaired in *Hdc* ^-/-^ macrophage clusters (**Figure 5D**). IFN-γ is known to exert antibacterial activity in macrophages by enhancing phagocytic activity (43, 44). To identify whether histamine signaling is important for IFN-γ in macrophages, total PMs and BMDMs were stimulated with LPS, and the expression level of IFN-γ was determined using a cytometric bead array. No IFN-γ expression was detected in LPS-stimulated *Hdc* ^-/-^ PMs or in the BMDMs culture medium (**Figure 5E**), suggesting that the histamine signal is critical for IFN-γ secretion. An *in vitro* phagocytosis assay using CFSE-labeled *L. monocytogenes* in PMs of 4-week-old and 1-year-old *Hdc* ^+/+^ and *Hdc* ^-/-^ mice showed that histamine deficiency resulted in impaired phagocytic activity, with significant reduction of thenumber of *L. monocytogenes* per PM in *Hdc* ^-/-^ mice than in *Hdc* ^+/+^ mice (**Figure 5F**).

**Figure 6:**
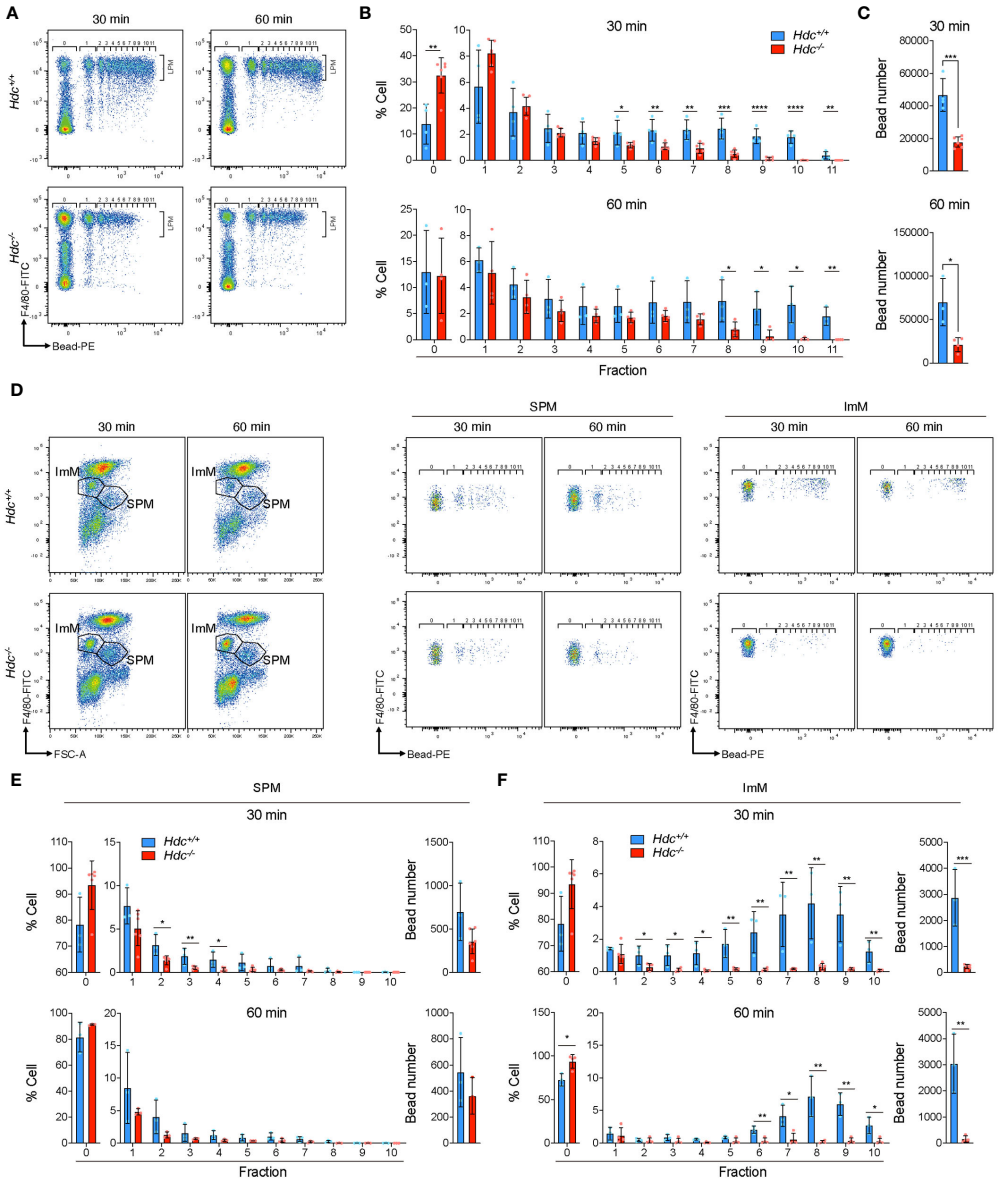
*In vivo* phagocytosis assay of peritoneal macrophages (PMs) from 4-week-old *Hdc* ^+/+^ and *Hdc* ^-/-^ mice using polystyrene beads. **(A)** Representative flow-cytometric images of F4/80^+^(FITC) Bead^+^(PE) large peritoneal macrophages (LPMs) at 30 min and 60 min. **(B)** Determination of the percentage of LPMs in each fraction. Fraction number indicates the number of beads in one LPM. **(C)** Total number of beads taken up. **(D)** Flow-cytometric gating strategy for immature macrophages (ImMs) and small peritoneal macrophages (SPMs). Representative flow-cytometric images of F4/80^mid^ Bead^+^(PE) ImM and F4/80^low^ Bead^+^(PE) SPM are shown. **(E, F)** Determination of the cell ratio in each fraction and calculation of the total uptaken bead number in SPMs **(E)** and ImMs **(F)**; n = 3 mice. Results are presented as the mean ± SD; *P < 0.05, **P < 0.01, ***P < 0.001, ****P < 0.0001.

To investigate whether histamine signaling deficiency also results in impaired phagocytic activity *in vivo*, fluorescence beads were intraperitoneally injected into 4-week-old *Hdc* ^+/+^ and *Hdc* ^-/-^ mice, and flow cytometric analysis was performed 30 and 60 min after bead injection (**Figure 6A**). Similar to the *in vitro* results, impaired phagocytic activity was detected in F4/80^high^ LPMs from *Hdc* ^-/-^ mice, with a significant reduction in the total phagocytosed bead number and the number of phagocytosed beads per cell in *Hdc* ^-/-^ than in *Hdc* ^+/+^ mice at both 30 min and 60 min (**Figures 6B, C**). We also compared the *in vivo* phagocytic activity of the SPM and ImM populations in *Hdc* ^+/+^ and *Hdc* ^-/-^ cells using flow cytometric analysis (**Figure 6D**). Histamine deficiency resulted in partially impaired phagocytic activity in SPMs and the phagocytic activity of ImMs from *Hdc* ^-/-^ mice was completely lost (**Figures 6E, F**). Taken together, these results indicated that histamine signal-mediated macrophage differentiation is important for the phagocytic ability of PMs.

### 3.5 Abnormally differentiated macrophages in *Hdc* ^-/-^ mice increase susceptibility to bacteria-mediated peritonitis

**Figure 7:**
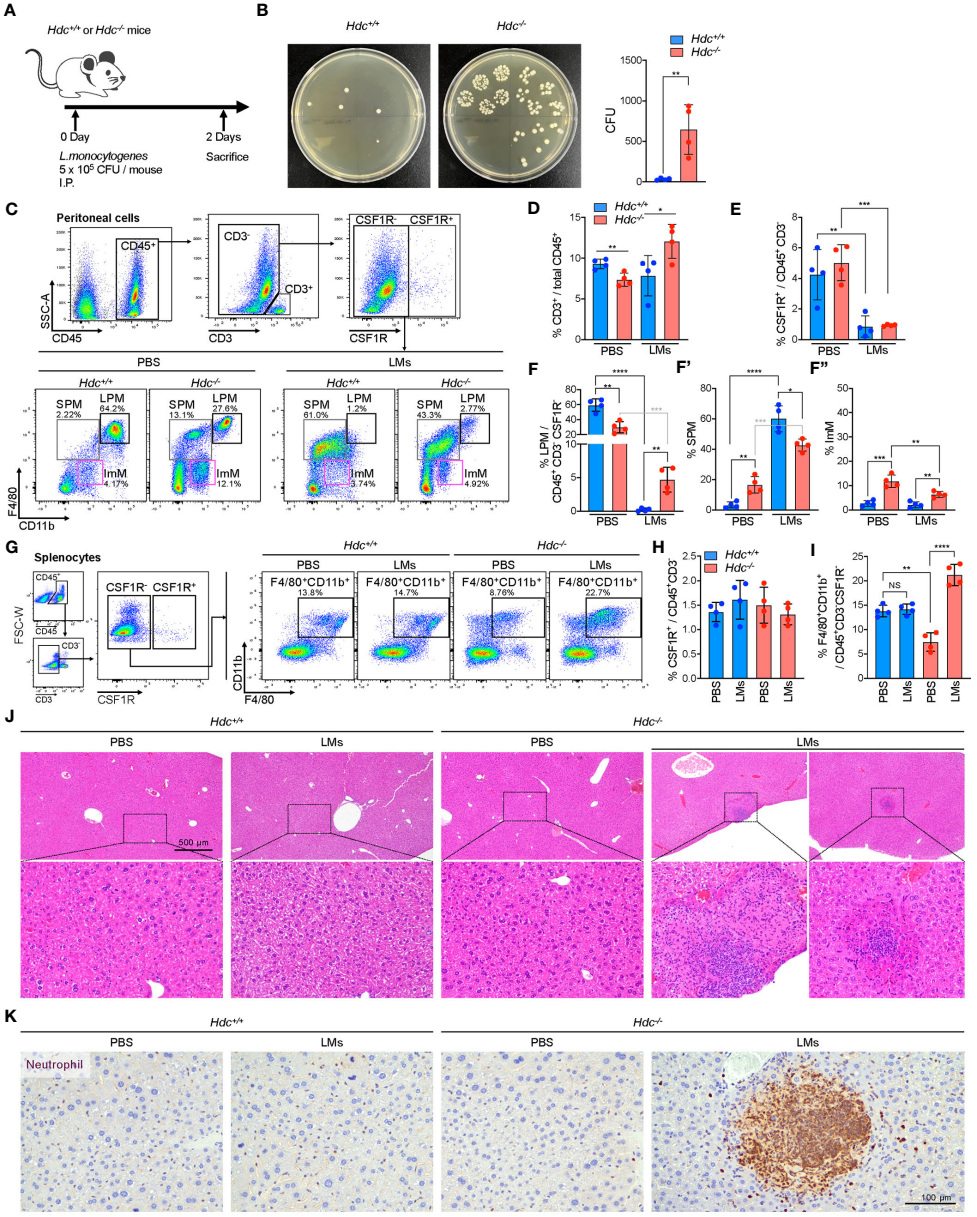
Establishment of an *in vivo L. monocytogenes* (LM)-mediated acute peritonitis model from 4-week-old *Hdc* ^+/+^ and *Hdc* ^-/-^ mice. **(A)** Scheme of the acute LMs infection model. **(B)** Left: representative brain heart infusion agar plate images for determination of the LM CFU from the peritoneal cavity 2 days after infection. Right: Expected number of LMs in the peritoneal cavity based on the colony count on brain heart infusion agar. **(C)** Flow cytometric gating strategy to characterize the changes of the peritoneal macrophage population after LMs infection. PBS control-treated or LMs-infected *Hdc* ^+/+^ and *Hdc* ^-/-^ mouse peritoneal cells were first gated on live (DAPI^-^), single, CD45^+^, CD3^-^, and CSF1R^-^ cells, followed by three overlapping gates as described in **Figure 1**. **(D–F)** Quantification of the proportion (%) of **(D)** CD3^+^ T cells among total CD45^+^ cells, **(E)** CSF1R^+^ cells among total CD45^+^CD3^-^ cells, and **(F)** large peritoneal macrophages (LPMs), small peritoneal macrophages (SPMs), and immature macrophages (ImMs) among total CD45^+^CD3^-^CSF1R^-^ cells. **(G)** Flow cytometric gating strategy of spleen macrophages. Total splenocytes were first gated on live (DAPI^-^), single, CD45^+^, CD3^-^, and CSF1R^-^ cells, and CD11b^+^F4/80^+^ macrophages were identified. **(H, I)** Quantification of the proportion (%) of **(H)** CSF1R^+^ cells among total CD45^+^CD3^-^ cells and **(I)** CD11b^+^F4/80^+^ macrophages among total CD45^+^CD3^-^CSF1R^-^ cells. **(J)** H&E-stained images of the liver of PBS-treated or LMs-infected *Hdc* ^+/+^ and *Hdc* ^-/-^ mice. **(K)** Images of liver sections immunohistochemically stained with anti-neutrophil antibody. n = 4 mice per group. Results are presented as the mean ± SD. *P < 0.05, **P < 0.01, ***P < 0.001, ****P < 0.0001.

Given the impact of histamine signaling deficiency on phagocytosis, we next questioned whether histamine-mediated macrophages differentiation is important for suppressing a bacterial infection *in vivo*. To explore this possibility, a mouse model of short-term peritonitis was established in which *Hdc* ^+/+^ and *Hdc* ^-/-^ mice were i.p. injected with *L. monocytogenes* (**Figure 7A**). Two days after infection, we found that the majority of the *L. monocytogenes* had been cleared in the peritoneal cavity of *Hdc* ^+/+^ mice, whereas a significant amount of live bacteria remained in the cavity of *Hdc* ^-/-^ mice (**Figure 7B**). Flow cytometry was then used to evaluate the dynamics of immune cells, especially macrophages, in the peritoneal cavity during infection (**Figure 7C**). The number of CD3^+^ T cells was reduced in *Hdc* ^-/-^ mice compared with that in *Hdc* ^+/+^ mice, but significantly increased after *L. monocytogenes* infection (**Figure 7D**). CSF1R^+^ macrophages dramatically reduced in response to *L. monocytogenes* infection in both *Hdc* ^+/+^ and *Hdc* ^-/-^ mice (**Figure 7E**). Notably, most of the LPMs disappeared in *Hdc* ^+/+^ mice following infection and only 10% of the injected *L. monocytogenes* remained (**Figure 7F**), indicating that *Hdc* ^+/+^ LPMs effectively participate in the clearance of invading bacteria and undergo activation-induced cell death. Reduced anti-bacterial activity was evident in *Hdc* ^-/-^ mice. The number of SPMs was higher in *Hdc* ^-/-^ than in *Hdc* ^+/+^ mice under the control (uninfected) condition, and this pattern reversed after *L. monocytogenes* infection (**Figure 7F**’). There was no change in the ImM population number in *Hdc* ^+/+^ mice following infection (**Figure 7F**’’).

*L. monocytogenes* is the main cause of listeriosis, which could lead to metastatic infection by spreading to various organs, including the spleen and liver through the circulation system (45). We therefore performed flow cytometric analysis of spleen macrophages in response to *L. monocytogenes* infection in *Hdc* ^+/+^ and *Hdc* ^-/-^ mice (**Figure 7G**). No change in the number of CSF1R^+^ macrophages was detected in both *Hdc* ^+/+^ and *Hdc* ^-/-^ mice (**Figure 7H**). The F4/80^+^CD11b^+^ spleen macrophage population significantly increased in the *L. monocytogenes*-infected *Hdc* ^-/-^ group, but there was no change in the number of *Hdc* ^+/+^ spleen macrophages (**Figure 7I**). Interestingly, H&E staining of the liver showed inflammatory foci only in the *L. monocytogenes*-infected *Hdc* ^-/-^ group (2 out of 4 mice; **Figure 7J**) and neutrophils were the most abundant cell population in inflammatory lesions (**Figure 7K**). In contrast, inflammatory foci in the liver were not observed in the *L. monocytogenes*-infected *Hdc* ^+/+^ group (**Figure 7J**). Since mice were sacrificed only two days after *L. monocytogenes* infection, it was difficult to observe consistent inflammatory foci in the liver. However, our data certainly showed more abundant inflammatory foci in *Hdc* ^-/-^ group after *L. monocytogenes* infection. Taken together, these results demonstrate that histamine deficiency-mediated impaired macrophage differentiation increases the susceptibility to *L. monocytogenes* infection as the PMs of *Hdc* ^-/-^ mice could not prevent the spread of *L. monocytogenes* to the spleen and liver, ultimately causing metastatic infection.

## 4 Discussion

Phagocytosis is the major biological process in the innate immune response, which maintains tissue homeostasis through the clearance of invading pathogens and apoptotic cells (46). The phagocytic activity of macrophages is required for the activation of histamine receptor signaling (28, 29). Therefore, histamine signaling is necessary for activating the host defense system in response to invading pathogens.

Using single-cell analysis of PMs, Lantz et al. (41) revealed the molecular profiles of LPMs and SPMs, and also demonstrated that MerTK was an important receptor for efferocytosis. This finding is consistent with the results of the present study demonstrating that the SPM cluster is characterized by low expression of *Adgre1*, but high expression of *Cd74.* In addition, scRNA-seq and flow cytometry analyses demonstrated reduced phagocytic activity of PMs, and increases in CCR2-, THBS1-, and IL-1α-positive macrophages in histamine signal-deficient PMs, which may increase the susceptibility to infectious diseases such as peritonitis. Our previous scRNA-seq analysis only showed functional defects in stomach-specific macrophage populations (31); however, the present scRNA-seq analysis of PMs in *Hdc* ^-/-^ mice revealed that histamine signal deficiency resulted in the generation of a new ImM population with impaired macrophagic activities, especially phagocytosis. Eosinophils also express CD11b; therefore, this newly identified ImM population may be eosinophils. Previous reports have shown that eosinophils are enriched in the peritoneal cavity under stimulating conditions such as in cases of infection or following thioglycollate treatment, whereas they are barely detected in the resting stage (47, 48). Our scRNA-seq and flow cytometry analyses were performed in resting conditions, and the ImM cluster did not express *Siglecf*, a key marker of eosinophils.

According to the gene expression pattern and UMAP location, the SPM population appeared to be related to monocytes, whereas the ImM population was more closely related to LPMs. Histamine has been shown to modulate H_2_ receptor–expressing hematopoietic stem cells (23), and our previous study showed that histamine-mediated stomach macrophage differentiation is required at the bone marrow cell stage (31). Although LPMs originate from the yolk sac and are maintained by the proliferation of tissue-resident macrophages, blood circulating bone marrow precursors can localize to the peritoneal cavity and differentiate into LPMs under specific conditions (49). Indeed, in the present study, *in vitro* treatment of PMs with histamine did not induce LPM differentiation; therefore, it is possible that bone marrow precursors continuously support the peritoneal Mac-pro population and histamine-deficient bone marrow precursors result in the generation of abnormal Mac-pro cells, thus interfering with their differentiation into ImMs and LPMs. Furthermore, the SPM population (which is supported by bone marrow precursors) was significantly increased in *Hdc* ^-/-^ mice compared to that in *Hdc* ^+/+^ mice. This suggests that the influx of bone marrow precursors increased due to the inability to maintain PMs under the histamine-deficient condition. By contrast, in the *in vivo L. monocytogenes* infection model, highly phagocytic *Hdc* ^+/+^ LPMs were found to actively participate in the clearance of invading bacteria, followed by activation-induced cell death. To fill this gap in the LPM population, the *Hdc* ^+/+^ bone marrow precursors produce more monocytes that differentiate into SPMs.

Further studies are needed to examine histamine-mediated macrophage differentiation into various types of myeloid cells under various environmental conditions. Although THBS1 is known as a GATA6-dependent LPM-specific factor (40), it has also been implicated in inflammation (50), and a myeloid-derived suppressor cell-like THBS1-macrophage subset was reported to be enriched in tumors (51). Our results demonstrated that histamine signal deficiency led to high expression of THBS1 in the ImM population, and the ImM cluster also showed upregulation of inflammation-related gene sets. Therefore, targeting the histamine signal pathway could be a potential novel anti-inflammatory therapeutic approach by inhibiting THBS1.

## Data availability statement

The original contributions presented in the study are publicly available. This data can be found here: https://www.ncbi.nlm.nih.gov/geo/, GSE232834.

## Ethics statement

The animal study was approved by IACUC of Yonsei University Health System. The study was conducted in accordance with the local legislation and institutional requirements.

## Author contributions

KK: Conceptualization, Data curation, Investigation, Project administration, Writing – original draft, Writing – review & editing, Formal Analysis, Methodology, Resources, Validation, Visualization. DP: Conceptualization, Data curation, Investigation, Visualization, Writing – original draft, Writing – review & editing. SC: Conceptualization, Data curation, Visualization, Writing – original draft, Writing – review & editing, Formal Analysis, Methodology, Resources, Software. YC: Data curation, Methodology, Visualization, Writing – review & editing, Validation. BL: Methodology, Validation, Visualization, Writing – review & editing, Investigation. HJ: Writing – review & editing. YuL: Writing – review & editing, Investigation, Validation, Visualization. YoL: Writing – review & editing, Methodology, Validation, Visualization. KN: Conceptualization, Data curation, Funding acquisition, Investigation, Project administration, Supervision, Writing – original draft, Writing – review & editing, Methodology.
