# Corrigendum: Role of histamine-mediated macrophage differentiation in clearance of metastatic bacterial infection

**DOI:** 10.3389/fimmu.2023.1341818

**Published:** 2023-11-28

**Authors:** Kwang H. Kim, Donghwan Park, Soo Young Cho, Yejin Cho, Buhyun Lee, Haengdueng Jeong, Yura Lee, Yourim Lee, Ki Taek Nam

**Affiliations:** ^1^ Department of Biomedical Sciences, Yonsei University College of Medicine, Seoul, Republic of Korea; ^2^ Department of Molecular and Life Science, Hanyang University College of Science and Convergence Technology, Ansan, Republic of Korea; ^3^ Department of Pathology, Seoul National University Hospital, Seoul, Republic of Korea

**Keywords:** histamine, macrophage differentiation, bacterial infection, single-cell RNA sequencing, peritoneal cells

In the published article, there was an error in the Funding statement. The National Research Foundation (NRF)’s funding from “the Ministry of Science & ICT(RS-2023-00241446)” should be added. The correct Funding statement appears below.

**FUNDING**

This research was supported by the Basic Science Research Program through the National Research Foundation of Korea (NRF) funded by the Ministry of Education (2017R1A6A3A04009690, 2022R1A2C3007850, 2022M3A9F3016364), the Ministry of Science & ICT (RS-2023-00241446) and the Korea Mouse Phenotyping Project (NRF-2016M3A9D5A01952416).

The authors apologize for this error and state that this does not change the scientific conclusions of the article in any way. The original article has been updated.

