# Supplementary material for: Role of histamine-mediated macrophage differentiation in clearance of metastatic bacterial infection
Source: Front Immunol. 2023 Nov 14;14:1290191. doi: 10.3389/fimmu.2023.1290191 (PMC10682073; doi:10.3389/fimmu.2023.1290191)
Supplement: Supplementary file 1 [file DataSheet_1.pdf]

Role of histamine-mediated macrophage differentiation in clearance of metastatic bacterial infection

Supplemental information

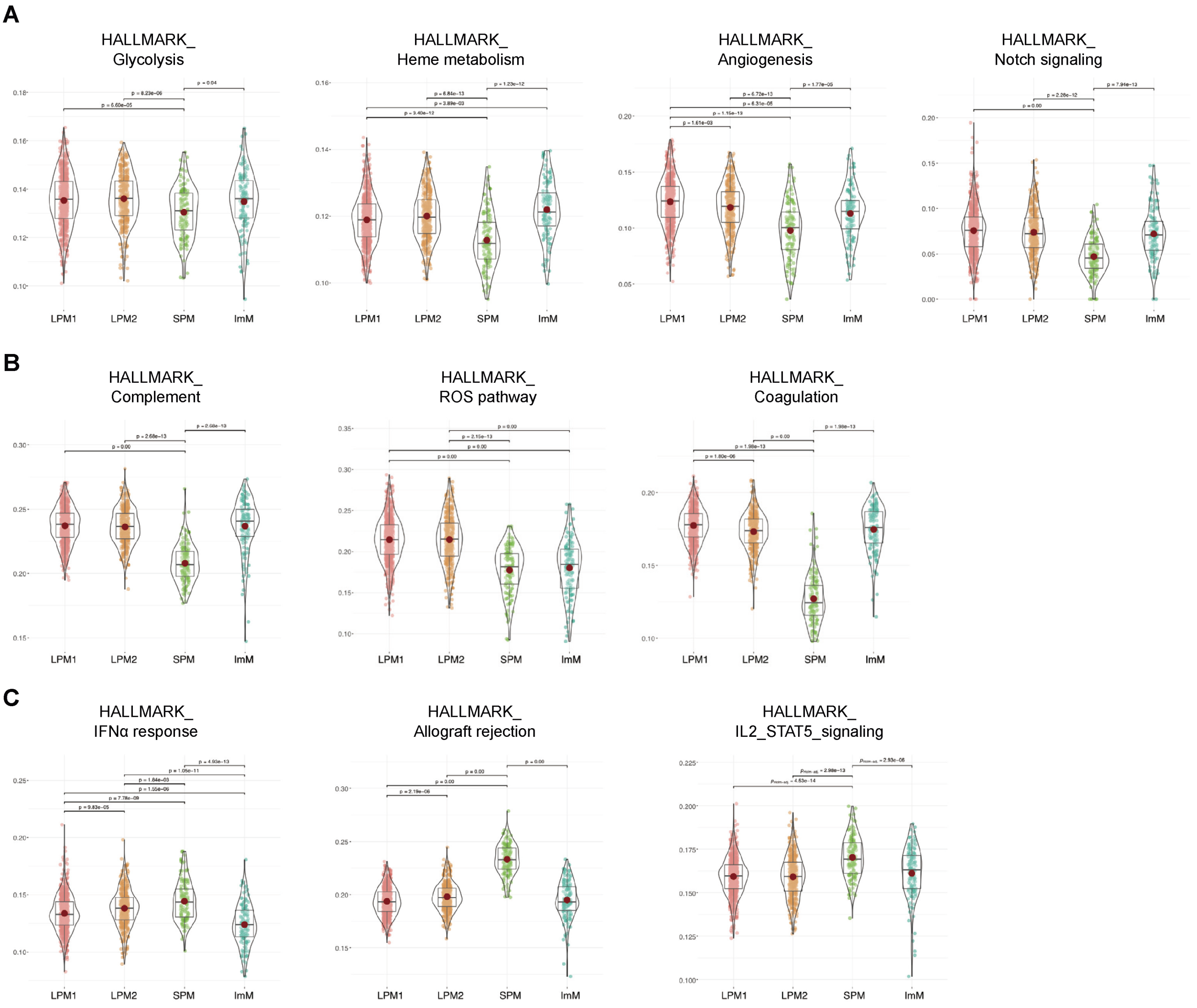

Supplementary figure S1. HALLMARK analysis in LPM1, LPM2, SPM, and ImM populations. (A–C) HALLMARK analysis of glycolysis, heme metabolism, angiogenesis, and Notch signaling (A); complement, ROS pathway, and coagulation (B); and the IFNα response, allograft rejection, and IL2–STAT5 signaling (C) of LPM1, LPM2, SPM, and ImM populations.

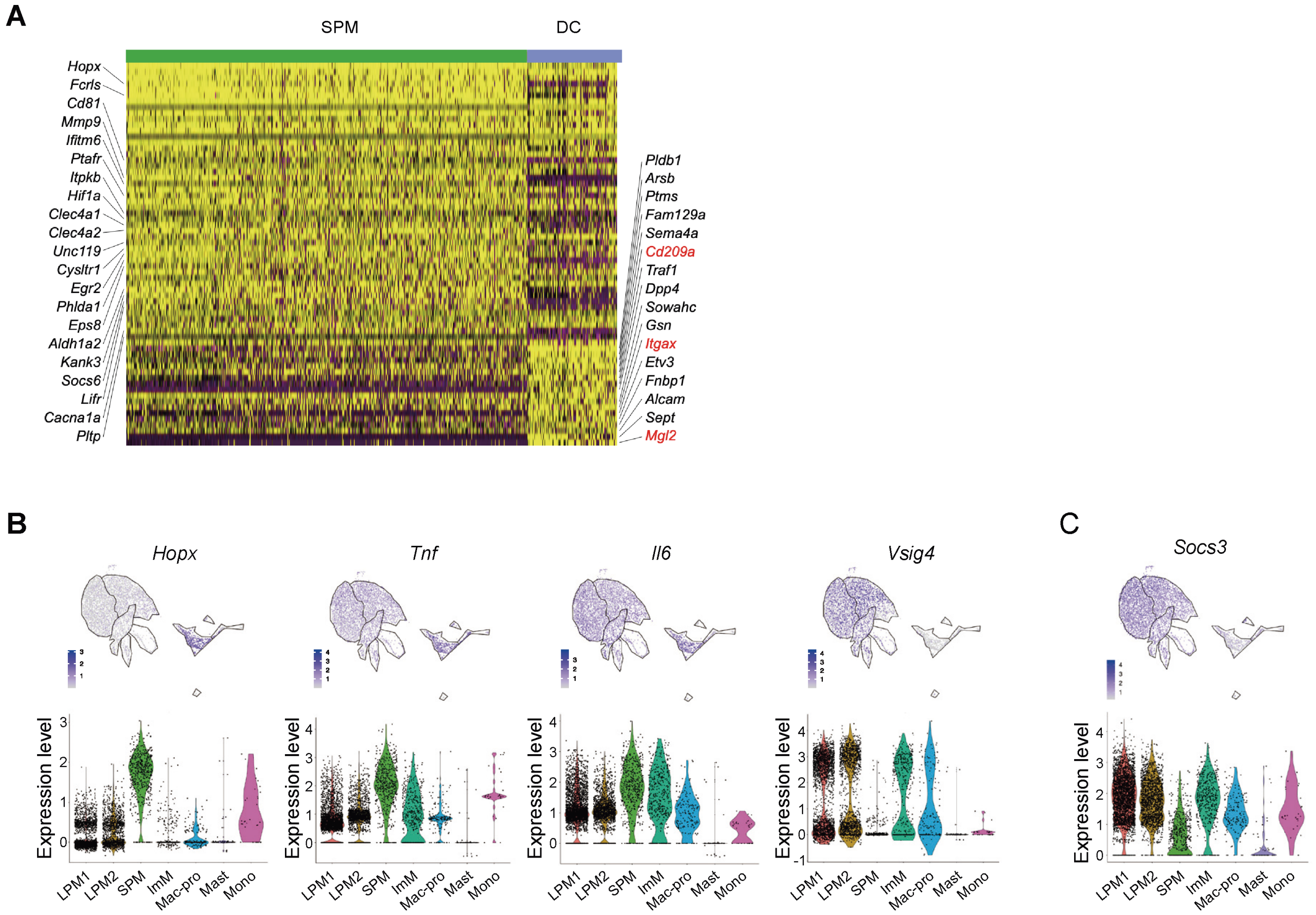

Supplementary figure S2. Phenotypic characterization of SPMs.
(A) Heat map of scaled expression of SPM and DC clusters. Genes with increased expression levels in SPM compared to DC and vice versa are highlighted on the left and right side of the heat map, respectively. DC marker genes are shown in red. (B) Specific genes differentially expressed in SPM defined on UMAP plots (top) and violin plots (bottom). Other populations are displayed on the x-axis; each dot represents a single cell. (C) Comparison of *Socs3* expression in LPM1, LPM2, SPM, ImM, Mac-pro, Mast, and Mono clusters presented as a UMAP plot (top) and violin plot (bottom).

Macrophage	Monocyte	B cell	NK cell	T cell	Dendritic cell	Proliferation	Mast cell
Cd68	*Csf1r*	*CD19*	*Fcgr3a*	*CD3d*	*Cd209a*	*Mki67*	*Cma1*
Mertk	*Fcgr3a*	*Ms4a1*	*Ncam1*	*CD3g*	*Itgax*	*Cdk1*	
Adgre1	*Cx3cr1*	*CD79a*	*Klrb1*	*CD32*	*Mgl2*	*Mcm6*	
Itgam	*Ccr2*	*CD79b*	*Klrc1*	*CD27*		*Ccnb2*	
Cd163		*Blnk*	*Klrd1*	*CD28*			
			Klrf1	*IL7r*			
			Klrk1				

Supplementary table S1. Marker genes expressed according to cell type.

의생명과학부/박*환/2023-10-20 18:19:24

Gene	myAUC	avg_diff	power	avg_logFC	pct.1	pct.2	p_val_adj	cluster
Ccl5	1	5.1995332	1	3.5963961	1	0.459	NA	8
Nkg7l	1	3.3428221	1	3.1277858	0.995	0.149	NA	8
Ms4a4b1	1	3.3369001	1	2.810328	0.99	0.271	NA	8
Ms4a6c	0.999	2.2088271	0.998	2.1366419	1	0.571	NA	14
Ccr2	0.994	2.4986782	0.988	2.4620539	1	0.292	NA	5
AW1120101	0.994	3.0665905	0.988	2.9557289	0.985	0.402	NA	8
Klrd11	0.984	2.7439457	0.968	2.5796864	0.965	0.192	NA	8
Clec4a11	0.984	1.5206809	0.968	1.4876442	1	0.442	NA	14
Gzma	0.982	4.605823	0.964	4.4468138	0.955	0.199	NA	8
Lst11	0.982	2.4506507	0.964	2.5058234	0.969	0.287	NA	14
H2-DMb1	0.98	1.7852939	0.96	1.7185342	0.978	0.339	NA	5
Il1b2	0.979	2.6567776	0.958	2.7875416	1	0.457	NA	14
Ltb4r1	0.977	1.5515278	0.954	1.4701487	0.93	0.191	NA	5
Hopx	0.976	1.6067706	0.952	1.4947318	0.956	0.413	NA	5
H2-Aa4	0.976	2.0995177	0.952	2.0692346	1	0.698	NA	11
Plxnd1	0.974	1.469981	0.948	1.4473662	0.947	0.245	NA	5
Cd3g	0.973	2.1733117	0.946	2.2029508	0.959	0.232	NA	7
Ms4a4b	0.969	2.7391285	0.938	2.4862816	0.956	0.264	NA	7
Ctla2a	0.966	2.8306539	0.932	2.7410561	0.936	0.244	NA	8
H2-DMb11	0.961	1.6973134	0.922	1.579	0.975	0.367	NA	11
Fcrls	0.96	1.8560556	0.92	1.8048362	0.948	0.274	NA	5
Tnip3	0.959	2.0830932	0.918	2.1737667	0.983	0.444	NA	5
Ncr1	0.959	2.5081113	0.918	2.4979905	0.95	0.084	NA	8
Plbd1	0.959	1.8257989	0.918	1.8594775	0.959	0.297	NA	11
Olfm11	0.955	1.5743001	0.91	1.5124024	0.967	0.359	NA	11
AF2517051	0.954	1.3884042	0.908	1.3690454	0.906	0.16	NA	14
Gadd45b	0.953	1.3966501	0.906	1.2938794	0.969	0.615	NA	14
H2-Ab13	0.952	1.6047867	0.904	1.5255679	1	0.688	NA	5
Cd79b1	0.95	1.6940487	0.9	1.3523457	1	0.502	NA	3
Ebf12	0.95	1.6845299	0.9	1.6296308	0.993	0.537	NA	4
Ccr24	0.949	1.9376507	0.898	1.9966859	1	0.329	NA	14
Mrc1	0.946	1.5507499	0.892	1.5701368	0.965	0.437	NA	5
Plekho12	0.946	1.3675247	0.892	1.3150436	0.969	0.543	NA	14
Cd79a1	0.945	1.6347432	0.89	1.3891846	0.999	0.536	NA	3
Napsa1	0.945	1.4222548	0.89	1.2473631	0.999	0.556	NA	3
Efhd2	0.945	1.1203113	0.89	1.1086908	0.98	0.66	NA	5
Vps37b	0.945	1.8708493	0.89	1.8700986	0.96	0.63	NA	8
Ms4a11	0.943	1.6098055	0.886	1.4071004	1	0.557	NA	3
Arsb	0.943	1.2503181	0.886	1.2226947	0.967	0.509	NA	11
Txk1	0.941	2.3998562	0.882	2.2610304	0.936	0.383	NA	8
Ptms	0.941	1.2355242	0.882	1.3854213	0.983	0.555	NA	11
Ms4a4c2	0.941	1.8970861	0.882	1.8289002	0.938	0.262	NA	14
Ebf11	0.94	1.5219643	0.88	1.1366631	0.999	0.505	NA	3
Clec4b1	0.94	1.2599623	0.88	1.2629254	0.895	0.336	NA	5
Skap1	0.939	1.6635197	0.878	1.5854993	0.883	0.043	NA	7
Ugcg	0.939	2.1842842	0.878	2.0626869	0.936	0.553	NA	8
Cd3d	0.938	2.0006792	0.876	2.0459747	0.921	0.1	NA	7
Scd11	0.936	1.2485135	0.872	1.0759264	0.978	0.515	NA	3
Plac81	0.935	1.5827142	0.87	1.5649972	1	0.547	NA	3
Fam129a1	0.935	0.9221834	0.87	1.0002139	0.934	0.25	NA	11
Olfm1	0.934	1.3966995	0.868	1.4848921	0.928	0.334	NA	5
H2-Aa3	0.933	1.4471717	0.866	1.2976587	1	0.684	NA	5
Il18r1	0.932	1.9098255	0.864	1.8212004	0.847	0.162	NA	8
Fcmr1	0.931	1.5569991	0.862	1.4839222	0.923	0.437	NA	4
Ccr23	0.931	1.5204213	0.862	1.504812	0.967	0.323	NA	11
Isg15	0.93	1.3794784	0.86	1.4073166	0.943	0.634	NA	5

Rab11fip1	0.93	1.0398186	0.86	0.9886362	0.888	0.386	NA	5
Tnip31	0.93	1.8796372	0.86	1.7115724	0.926	0.469	NA	11
Fgd21	0.928	1.0629514	0.856	0.9415638	0.962	0.358	NA	3
Ccdc88a	0.925	1.1819298	0.85	1.089046	0.954	0.645	NA	5
Cd28	0.925	1.6599676	0.85	1.7206471	0.87	0.192	NA	7
Thy1	0.924	1.3135128	0.848	1.4058477	0.86	0.083	NA	7
Efhd21	0.924	1.0982582	0.848	1.1462473	0.983	0.674	NA	11
Ccr22	0.923	1.9135151	0.846	1.5104035	0.911	0.319	NA	8
Ccr7	0.922	1.8752828	0.844	1.8282278	0.918	0.392	NA	4
Cd81	0.922	1.2826918	0.844	0.9904034	0.941	0.561	NA	5
Klre1	0.922	1.8431485	0.844	1.8201956	0.842	0.036	NA	8
Pid1	0.92	1.0725417	0.84	1.0883026	0.989	0.579	NA	5
Mcm5	0.92	0.7448545	0.84	0.7419296	0.912	0.316	NA	10
Sema4a1	0.919	0.7774954	0.838	0.7097937	0.876	0.352	NA	11
Cd21	0.918	1.2824505	0.836	1.219511	0.998	0.505	NA	3
Ccdc109b	0.918	1.0326039	0.836	1.1065304	0.948	0.616	NA	5
Klrk11	0.918	2.0163049	0.836	1.8919044	0.856	0.304	NA	8
Pou2f21	0.916	1.2045809	0.832	1.1413082	0.997	0.622	NA	3
Stmn11	0.916	1.7528943	0.832	1.6652648	0.856	0.222	NA	12
Mmp9	0.915	2.0647085	0.83	1.9919974	0.829	0.368	NA	5
Hal	0.914	1.2621513	0.828	1.2268995	0.973	0.492	NA	1
Ifitm62	0.914	1.1003485	0.828	1.1191089	0.974	0.663	NA	5
Slamf9	0.914	1.0949223	0.828	1.1043432	0.913	0.395	NA	5
Cd209a	0.914	2.3610932	0.828	2.314627	0.884	0.33	NA	11
Cd9	0.912	1.1110927	0.824	1.1482955	0.999	0.585	NA	0
Ptafr	0.912	0.949609	0.824	0.9863189	0.936	0.502	NA	5
Stmn1	0.912	1.6016565	0.824	1.6696339	0.899	0.217	NA	10
Mcm3	0.912	0.6756381	0.824	0.663884	0.95	0.378	NA	10
Bcl2a1a	0.911	1.055701	0.822	1.0627185	0.895	0.31	NA	5
Basp1	0.911	2.6154932	0.822	2.5382516	0.877	0.387	NA	13
Traf12	0.911	1.1226415	0.822	1.1696854	0.969	0.524	NA	14
Ifng1	0.91	1.9059624	0.82	1.7084128	0.748	0.051	NA	8
Tmem176b	0.909	1.2967169	0.818	1.3509045	0.948	0.477	NA	5
Il1b1	0.909	2.1079408	0.818	2.0950349	0.909	0.453	NA	11
Mcm51	0.909	0.892052	0.818	0.8755487	0.876	0.32	NA	12
Abhd17b1	0.908	1.0964148	0.816	0.9608593	0.976	0.556	NA	3
Marcks1	0.908	1.2430199	0.816	1.1835116	0.95	0.609	NA	11
Traf11	0.908	1.3078666	0.816	1.1101168	0.917	0.521	NA	11
Clec12a	0.908	1.1898813	0.816	1.1605876	0.844	0.336	NA	14
Ms4a6b2	0.908	1.1892912	0.816	1.0682459	0.938	0.529	NA	14
Itpkb	0.907	0.9271655	0.814	0.7937882	0.926	0.548	NA	5
Siglecg1	0.906	1.0587516	0.812	0.983007	0.967	0.499	NA	3
Prg4	0.905	1.1725359	0.81	1.1970155	1	0.698	NA	0
Hif1a	0.904	0.9639821	0.808	0.9339823	0.972	0.664	NA	5
Clec4a1	0.904	0.9357763	0.808	0.9079335	0.884	0.418	NA	5
Dpp4	0.904	0.8347215	0.808	0.7681335	0.884	0.321	NA	11
Mcm31	0.903	0.9123323	0.806	0.8610238	0.887	0.383	NA	12
Ccdc88a1	0.902	1.0726915	0.804	1.1207601	0.959	0.658	NA	11
Clec4a2	0.901	1.1228504	0.802	1.1736098	0.913	0.354	NA	5
Pip4k2a1	0.9	0.9067288	0.8	0.9676289	0.967	0.603	NA	11
Plbd11	0.9	1.3794319	0.8	1.314868	0.875	0.303	NA	14
Ly6c1	0.9	1.0053278	0.8	0.9833339	0.812	0.086	NA	14
Naaa	0.899	1.0157364	0.798	0.909435	0.869	0.439	NA	5
Ly6c2	0.899	1.7180064	0.798	1.6602225	0.812	0.084	NA	14
Bcl2a1d	0.898	0.9872653	0.796	0.960172	0.917	0.551	NA	11
Unc119	0.897	0.8678225	0.794	0.9511199	0.947	0.641	NA	5
Smc2	0.897	0.914113	0.794	0.9159885	0.925	0.383	NA	10

Cysltr1	0.896	0.9883421	0.792	0.9995682	0.891	0.42	NA	5
Cd79a2	0.894	1.2659653	0.788	1.2251752	0.995	0.566	NA	4
Egr2	0.894	1.2269156	0.788	1.3577855	0.934	0.597	NA	5
Ms4a6d2	0.894	1.5237928	0.788	1.4860778	0.906	0.572	NA	14
Aif1	0.894	1.4406278	0.788	1.4179231	0.844	0.307	NA	14
Il18rap1	0.893	1.7387662	0.786	1.6867372	0.817	0.244	NA	8
Naaa1	0.893	1.6372262	0.786	1.6304711	0.901	0.457	NA	11
Pla2g7	0.892	1.1093491	0.784	1.0487122	0.997	0.618	NA	0
Ms4a1	0.892	1.3027651	0.784	1.2097307	0.995	0.548	NA	2
Fgd2	0.892	0.8613285	0.784	0.5902372	0.838	0.365	NA	2
Mzb11	0.892	1.2758152	0.784	1.2370037	0.988	0.415	NA	3
Cd191	0.892	0.8426343	0.784	0.8775582	0.955	0.472	NA	3
Il2rb	0.892	1.5378518	0.784	1.4447248	0.772	0.133	NA	8
Sowahc	0.892	1.0337262	0.784	0.9711538	0.942	0.595	NA	11
2700094K13Rik1	0.892	0.9841882	0.784	0.9032012	0.959	0.58	NA	12
AI839979	0.892	0.6975534	0.784	0.6917113	0.75	0.14	NA	14
Mzb1	0.891	1.1817339	0.782	1.0969456	0.965	0.406	NA	2
H2-Aa2	0.891	1.2766778	0.782	1.0614267	0.997	0.683	NA	4
Il7r	0.891	1.3937048	0.782	1.5231321	0.816	0.069	NA	7
Eomes	0.891	1.7205764	0.782	1.6646526	0.762	0.061	NA	8
Ralgps21	0.89	0.9302133	0.78	0.7584546	0.926	0.408	NA	3
Spry2	0.89	1.8965452	0.78	1.9714242	0.881	0.346	NA	8
Gimap43	0.89	1.5183748	0.78	1.4176618	0.901	0.517	NA	8
Mcm71	0.89	0.8185435	0.78	0.8121719	0.835	0.288	NA	12
Phlda1	0.889	1.428892	0.778	1.3891792	0.919	0.599	NA	5
Atad2	0.889	0.8351698	0.778	0.7507742	0.899	0.395	NA	10
Clec4a3	0.888	0.8014969	0.776	0.8120558	0.884	0.417	NA	5
Satb12	0.888	1.7112856	0.776	1.6364875	0.851	0.377	NA	8
Batf3	0.887	0.9103239	0.774	0.8495954	0.779	0.185	NA	5
Eps8	0.887	0.9491911	0.774	0.8160976	0.921	0.577	NA	5
Plac8	0.886	1.4116411	0.772	1.379146	0.993	0.538	NA	2
Zbtb321	0.886	0.7605646	0.772	0.7730237	0.913	0.376	NA	3
Il1b	0.886	1.8671959	0.772	1.7488159	0.842	0.436	NA	5
Aldh1a2	0.886	0.8377335	0.772	0.8348698	0.788	0.145	NA	5
Mcm4	0.886	0.4613408	0.772	0.4792794	0.893	0.341	NA	10
Gsn1	0.886	1.0620959	0.772	0.9382295	0.826	0.257	NA	11
Kank3	0.885	0.9003184	0.77	0.8087599	0.799	0.284	NA	5
Socs6	0.885	0.8173992	0.77	0.7306721	0.794	0.41	NA	5
Fcna1	0.884	1.1193566	0.768	1.2207332	0.999	0.659	NA	1
Ets13	0.883	1.1744265	0.766	1.2559481	0.959	0.488	NA	7
Itgax	0.883	0.8048369	0.766	0.7850982	0.777	0.046	NA	11
Serpinb1a	0.882	0.965877	0.764	0.941995	0.959	0.547	NA	0
Uhrf11	0.882	0.7654467	0.764	0.7667401	0.814	0.197	NA	12
Clec4a31	0.882	1.0802467	0.764	1.0476531	0.906	0.441	NA	14
Ltb4r11	0.881	1.1130689	0.762	1.0966603	0.876	0.224	NA	11
Tnip32	0.881	1.1748498	0.762	1.159313	0.906	0.473	NA	14
1110059E24Rik	0.88	0.7859822	0.76	0.5634405	0.889	0.494	NA	3
Cd3e	0.88	1.6494059	0.76	1.5457072	0.825	0.227	NA	7
Dut1	0.88	1.0376433	0.76	1.0249606	0.876	0.356	NA	12
Ms4a6b1	0.879	1.5801717	0.758	1.5453548	0.908	0.517	NA	7
Zyx1	0.879	0.8887931	0.758	0.8785067	1	0.65	NA	11
Lifr	0.878	0.8554647	0.756	0.7626208	0.855	0.496	NA	5
Sepp11	0.877	0.9997233	0.754	1.0849856	0.999	0.646	NA	1
Etv3	0.877	1.1630871	0.754	1.1218606	0.893	0.509	NA	11
Gmnn1	0.877	0.7392898	0.754	0.6665811	0.825	0.393	NA	12
Gm51501	0.877	0.8156457	0.754	0.832098	0.844	0.306	NA	14
Cd14	0.876	1.0532	0.752	0.8859261	1	0.682	NA	0

Pmf11	0.876	0.8300208	0.752	0.7770837	0.89	0.373	NA	3
Ltb2	0.876	1.3059174	0.752	1.1966254	0.979	0.566	NA	4
Zyx	0.876	0.8052585	0.752	1.0623056	0.972	0.635	NA	5
Pip4k2a	0.875	0.8195678	0.75	0.7509194	0.895	0.591	NA	5
Txk	0.875	1.422564	0.75	1.32911	0.838	0.379	NA	7
Cks1b	0.875	0.8883592	0.75	0.8371593	0.862	0.388	NA	10
Ltb	0.874	1.0775794	0.748	1.0999421	0.966	0.529	NA	2
Pkig	0.874	0.7798953	0.748	0.8106568	0.964	0.631	NA	3
Itga62	0.874	0.9213757	0.748	0.9391975	0.974	0.644	NA	6
Nupr1	0.873	0.9768301	0.746	0.8982909	0.992	0.472	NA	0
H2-Ab12	0.873	1.0685834	0.746	0.8948817	0.997	0.686	NA	4
Lig11	0.873	0.8531568	0.746	0.8283257	0.825	0.327	NA	12
Marcks	0.872	0.9881942	0.744	1.0423013	0.915	0.595	NA	5
Fnbp1	0.872	0.8952851	0.744	0.9866559	0.959	0.636	NA	11
Sowahc1	0.872	1.0913572	0.744	1.1381196	0.938	0.598	NA	14
Ets12	0.871	1.2085965	0.742	1.1975756	0.943	0.476	NA	4
Dusp5	0.871	1.5527189	0.742	1.3946277	0.835	0.487	NA	7
Rgs10	0.87	0.8587521	0.74	0.8652401	0.997	0.567	NA	0
Top2a	0.87	1.4020894	0.74	1.3936826	0.855	0.385	NA	10
Uhrf1	0.87	0.5521312	0.74	0.5628508	0.824	0.193	NA	10
Rrm11	0.87	0.6151018	0.74	0.5690548	0.794	0.317	NA	12
Ccl24	0.869	1.2012817	0.738	1.0285474	1	0.618	NA	0
Cbr2	0.869	0.7345181	0.738	0.7078923	0.867	0.399	NA	1
Gm83691	0.869	1.5093297	0.738	1.4706665	0.921	0.469	NA	4
Cacna1a	0.869	0.8479928	0.738	0.7390133	0.805	0.477	NA	5
Ets14	0.869	1.1950459	0.738	1.1256773	0.926	0.494	NA	8
Alcam1	0.869	0.8321271	0.738	0.8495493	0.967	0.541	NA	11
Icam2	0.868	0.9757423	0.736	0.9476265	0.999	0.622	NA	0
Cd79b	0.868	1.1788411	0.736	1.1359504	0.996	0.492	NA	2
Pltp2	0.868	0.901504	0.736	0.905175	0.996	0.689	NA	5
Chaf1a	0.868	0.2749918	0.736	0.3045802	0.818	0.297	NA	10
Bcl2a1d1	0.868	0.8400658	0.736	0.8349985	0.875	0.555	NA	14
Spon1	0.868	0.2647921	0.736	0.2524848	0.594	0.061	NA	14
Snn1	0.867	0.730742	0.734	0.7379338	0.925	0.402	NA	3
Mki67	0.867	1.4568086	0.734	1.4996525	0.906	0.51	NA	10
Nasp1	0.867	0.7188681	0.734	0.7251421	0.918	0.419	NA	12
Acp5	0.866	0.7842787	0.732	0.8015626	0.909	0.298	NA	3
4930523C07Rik1	0.866	0.8980248	0.732	0.7938222	0.979	0.388	NA	3
Blnk1	0.866	0.6917888	0.732	0.5711922	0.875	0.341	NA	3
Dctpp11	0.866	0.6815417	0.732	0.6263496	0.897	0.48	NA	12
Mgst1	0.865	0.7893957	0.73	0.7336491	0.98	0.564	NA	0
Dusp51	0.865	1.5532079	0.73	1.5773262	0.886	0.49	NA	8
03-Sep	0.865	0.8133874	0.73	0.7902691	0.744	0.004	NA	11
Cyp4f18	0.864	1.0179793	0.728	1.0115085	0.831	0.329	NA	3
Plxnd11	0.864	0.7689088	0.728	0.7780504	0.843	0.277	NA	11
Tmem176a	0.862	1.2345048	0.724	1.2310171	0.877	0.437	NA	5
Pid11	0.862	0.9713154	0.724	0.9654186	0.959	0.597	NA	11
Garnl3	0.861	0.6852294	0.722	0.5954125	0.813	0.336	NA	0
Hck	0.861	0.7785719	0.722	0.7710607	0.895	0.502	NA	3
Pou2af11	0.861	0.6606505	0.722	0.6839559	0.932	0.45	NA	3
Cx3cr11	0.861	1.2365645	0.722	1.3226211	0.812	0.181	NA	14
Naaa2	0.861	0.8986654	0.722	0.839089	0.875	0.461	NA	14
Klrb1f	0.86	1.367582	0.72	1.2989374	0.718	0.065	NA	8
Cks1b1	0.86	1.015305	0.72	0.9427644	0.784	0.392	NA	12
Pid12	0.859	1.1501727	0.718	1.2019206	0.906	0.6	NA	14
Scd1	0.858	0.8914411	0.716	0.7633605	0.907	0.516	NA	2
Tmem123	0.858	0.8817596	0.716	0.9376423	0.969	0.665	NA	3

AU020206	0.858	0.6992248	0.716	0.6648525	0.888	0.49	NA	3
Fcer2a	0.858	1.417618	0.716	1.6891758	0.837	0.197	NA	4
Coro1a7	0.858	0.9189789	0.716	0.9674455	1	0.639	NA	14
Rnf125	0.857	0.9256517	0.714	0.8789971	0.708	0.147	NA	7
Clec4b11	0.857	1.1675535	0.714	1.1406517	0.818	0.362	NA	11
Cbx51	0.857	0.5182479	0.714	0.5041672	0.835	0.363	NA	12
Fcmr	0.856	0.9468406	0.712	1.0390062	0.867	0.402	NA	2
4930523C07Rik	0.856	0.8528586	0.712	0.9172135	0.956	0.378	NA	2
H2-DMb2	0.856	0.9095697	0.712	0.8147979	0.955	0.429	NA	2
Gimap4	0.856	0.812756	0.712	0.7172198	0.882	0.467	NA	2
Gm436031	0.856	0.7002665	0.712	0.6142679	0.916	0.416	NA	3
Tmpo1	0.856	0.7773686	0.712	0.7060252	0.907	0.577	NA	12
Selp	0.855	0.9188643	0.71	0.9121321	0.998	0.508	NA	0
Hvcn1	0.855	1.3290737	0.71	1.2176396	0.809	0.285	NA	4
Cd79b2	0.855	1.146394	0.71	1.1936219	0.988	0.535	NA	4
Lig1	0.855	0.6882443	0.71	0.6441139	0.824	0.323	NA	10
Fchsd2	0.854	1.2557605	0.708	1.2475047	0.808	0.364	NA	4
Ecm1	0.853	0.8346795	0.706	0.8474808	0.999	0.575	NA	0
Rasgrp1	0.853	0.7594185	0.706	0.6397899	0.913	0.499	NA	3
Flnb	0.852	0.7858878	0.704	0.7786806	0.994	0.572	NA	0
Pax5	0.852	0.6851041	0.704	0.7589195	0.906	0.454	NA	2
Rrm1	0.852	0.7565138	0.704	0.7158788	0.83	0.314	NA	10
Mgl2	0.852	2.110962	0.704	2.2647459	0.826	0.375	NA	11
Mcm21	0.852	0.6792918	0.704	0.6863736	0.835	0.419	NA	12
Mgst2	0.851	0.524112	0.702	0.4400569	0.746	0.402	NA	3
Ass11	0.851	0.6354716	0.702	0.582442	0.845	0.321	NA	12
Ctsl	0.85	0.9006442	0.7	0.938694	0.999	0.633	NA	0
Mcemp1	0.85	0.785786	0.7	0.8088349	0.997	0.558	NA	0
Folr2	0.85	0.7046415	0.7	0.7001237	0.792	0.292	NA	1
Coro1a	0.85	0.9507536	0.7	0.8649031	0.993	0.583	NA	2
Pax51	0.85	0.7544805	0.7	0.6795786	0.943	0.459	NA	3
Klrc21	0.85	1.2799392	0.7	1.256387	0.668	0.019	NA	8
Lmnb11	0.85	0.7982048	0.7	0.7689296	0.814	0.396	NA	12
Tmem176b2	0.85	1.1160461	0.7	1.2020975	0.906	0.502	NA	14
Napsa	0.849	0.9508672	0.698	0.940912	0.96	0.552	NA	2
Cd19	0.849	0.8327473	0.698	0.7150172	0.865	0.475	NA	2
Slc2a6	0.849	0.6643125	0.698	0.5700328	0.74	0.319	NA	5
Nkg7	0.849	1.9689959	0.698	1.5290652	0.778	0.146	NA	7
Hmgn2	0.849	0.7683119	0.698	0.7516354	0.893	0.405	NA	10
Atad21	0.849	0.6224817	0.698	0.6242286	0.845	0.399	NA	12
Pou2f2	0.848	0.9484963	0.696	0.941182	0.983	0.616	NA	2
Fcrla1	0.848	0.8416435	0.696	0.7951859	0.921	0.462	NA	3
Atp1b1	0.848	1.2949089	0.696	1.2121658	0.698	0.21	NA	8
Racgap1	0.848	0.6705093	0.696	0.6706026	0.805	0.314	NA	10
Lmnb1	0.848	0.5984941	0.696	0.6262787	0.855	0.393	NA	10
Gata6	0.847	0.6348052	0.694	0.4892165	0.836	0.364	NA	0
Fasl	0.847	1.394454	0.694	1.3785851	0.718	0.029	NA	8
1700025G04Rik2	0.847	0.6715365	0.694	0.5713069	0.752	0.173	NA	11
Clec4d	0.846	0.84867	0.692	0.7734442	0.998	0.593	NA	0
F5	0.845	0.8682459	0.69	0.9670728	1	0.583	NA	0
F10	0.845	0.838758	0.69	0.8226602	0.965	0.437	NA	0
Pafah1b3	0.845	0.8239294	0.69	0.7778544	0.91	0.421	NA	3
Il1rn	0.845	1.0629634	0.69	1.0987707	0.84	0.449	NA	5
Cd8b1	0.845	1.2151797	0.69	1.2129601	0.702	0.006	NA	7
Mcm7	0.845	0.5295676	0.69	0.5775553	0.868	0.284	NA	10
Gm5150	0.845	0.9879894	0.69	0.9522473	0.777	0.302	NA	11
Dusp52	0.845	0.6578617	0.69	0.7498681	0.893	0.494	NA	11

Spib1	0.844	0.6893101	0.688	0.7015078	0.895	0.386	NA	3
Alcam	0.844	0.6588313	0.688	0.688388	0.895	0.526	NA	5
Itga6	0.843	0.853518	0.686	0.9375747	1	0.516	NA	0
Cd22	0.843	0.698205	0.686	0.6862428	0.874	0.396	NA	3
Pax52	0.843	1.1152514	0.686	1.0783366	0.875	0.494	NA	4
AW112010	0.843	1.6309982	0.686	1.2906498	0.863	0.399	NA	7
Cd5l3	0.843	1.0317925	0.686	0.8924456	0.987	0.591	NA	10
Ear21	0.843	0.7805625	0.686	0.7562501	0.694	0.104	NA	11
Slc15a3	0.843	0.8698958	0.686	0.8982203	0.938	0.576	NA	14
Slc16a31	0.843	0.4585393	0.686	0.3810774	0.75	0.329	NA	14
Ednrb2	0.842	0.8098546	0.684	0.8796918	0.985	0.684	NA	6
Ly6d	0.841	0.9913798	0.682	1.0444796	0.811	0.231	NA	2
H2-DMb21	0.841	0.7483045	0.682	0.7551976	0.975	0.438	NA	3
Irf41	0.841	0.5427339	0.682	0.4243858	0.842	0.404	NA	3
Ndnf	0.841	0.7253987	0.682	0.7372009	0.711	0.14	NA	5
Rap2a	0.841	0.7617267	0.682	0.7902139	0.86	0.46	NA	11
Pfkp1	0.841	0.7675229	0.682	0.7490439	0.893	0.504	NA	11
Gm12840	0.84	0.6563519	0.68	0.8538612	0.97	0.6	NA	0
Siglecg	0.84	0.8083088	0.68	0.7518761	0.906	0.498	NA	2
Tiam11	0.84	0.6286341	0.68	0.5667182	0.901	0.473	NA	11
Skap11	0.84	0.4514712	0.68	0.4280706	0.739	0.068	NA	15
Prdx4	0.839	0.699398	0.678	0.6449152	0.976	0.571	NA	0
Zcwpw1	0.839	0.8491715	0.678	1.0355259	0.808	0.39	NA	3
H2-Aa1	0.839	1.0216683	0.678	0.7015069	1	0.661	NA	3
Tnfrsf13b	0.839	0.5522449	0.678	0.4912612	0.883	0.522	NA	3
Svil	0.839	0.7613801	0.678	0.7907215	0.902	0.553	NA	5
Fgr	0.839	0.7470665	0.678	0.7549469	0.89	0.553	NA	5
Mcm61	0.839	0.778801	0.678	0.744919	0.784	0.326	NA	12
Msr12	0.838	0.7752265	0.676	0.6805813	1	0.693	NA	10
Gmnn	0.838	0.6204969	0.676	0.6168586	0.843	0.39	NA	10
Naga	0.838	0.9408242	0.676	0.9232958	0.86	0.557	NA	11
Tnfsf9	0.837	0.9202778	0.674	0.9234413	0.802	0.45	NA	11
Ltbp1	0.836	0.7904821	0.672	0.8600861	0.971	0.483	NA	0
Ggh	0.836	0.526945	0.672	0.4100947	0.913	0.475	NA	0
Blk1	0.836	0.6330034	0.672	0.5766613	0.896	0.471	NA	3
Tbxas1	0.836	0.7648691	0.672	0.739089	0.891	0.493	NA	5
Ctsw1	0.836	1.5855469	0.672	1.5345962	0.733	0.115	NA	8
Serpinb9	0.836	1.5725152	0.672	1.5290585	0.703	0.248	NA	8
H2-Ab1	0.835	0.872058	0.67	0.5748108	0.987	0.659	NA	2
Tnfrsf18	0.835	1.4823291	0.67	1.3603035	0.752	0.107	NA	7
Cd8a	0.835	0.5572022	0.67	0.6201687	0.683	0.004	NA	7
Tipin	0.835	0.4190068	0.67	0.4380133	0.912	0.463	NA	10
Timp2	0.834	0.9256846	0.668	0.8774239	1	0.639	NA	0
Plekho11	0.834	0.7660613	0.668	0.8172404	0.901	0.54	NA	11
Chn21	0.834	0.4227221	0.668	0.4687657	0.769	0.225	NA	11
Tipin1	0.833	0.7038327	0.666	0.6514843	0.825	0.466	NA	12
Arl5c	0.832	0.7035322	0.664	0.5551103	0.73	0.298	NA	3
Fgl22	0.832	0.6922654	0.664	0.8807522	0.826	0.319	NA	11
Wnt11	0.832	0.7448869	0.664	0.7065578	0.686	0.107	NA	11
Ptprcap	0.831	0.6768213	0.662	0.7288815	0.919	0.411	NA	2
Plekho1	0.831	0.687926	0.662	0.6615058	0.856	0.526	NA	5
Gimap32	0.831	1.1423079	0.662	1.3685282	0.902	0.517	NA	7
Nasp	0.831	0.5538359	0.662	0.5458242	0.918	0.415	NA	10
Dnmt1	0.831	0.6014877	0.662	0.5457093	0.849	0.397	NA	10
Dapk1	0.83	0.7119086	0.66	0.6899824	0.867	0.514	NA	5
Ltb3	0.83	1.22581	0.66	1.126956	0.937	0.579	NA	7
Klrc11	0.83	1.5841613	0.66	1.461632	0.663	0.058	NA	8

Lck1	0.83	1.3169708	0.66	1.2893904	0.728	0.255	NA	8
Batf31	0.83	0.7440446	0.66	0.7624594	0.777	0.211	NA	11
Lrg1	0.829	0.8345437	0.658	0.8333227	0.966	0.552	NA	0
Anxa1	0.829	0.7257398	0.658	0.7465962	0.991	0.606	NA	0
Blnk	0.829	0.6615476	0.658	0.5699403	0.774	0.346	NA	2
H2-Ab11	0.829	0.8674076	0.658	0.5352767	0.998	0.665	NA	3
Pbk	0.829	0.5600635	0.658	0.6123325	0.748	0.125	NA	10
Il1rl1	0.829	0.6360163	0.658	0.6017194	0.769	0.327	NA	11
Olfm12	0.829	0.9205046	0.658	0.8733453	0.812	0.365	NA	14
Gpr18	0.828	0.4586345	0.656	0.3674446	0.75	0.353	NA	3
Chn2	0.828	0.606098	0.656	0.6114213	0.707	0.204	NA	5
Tmpo	0.828	0.8483703	0.656	0.8481883	0.912	0.575	NA	10
Mcm2	0.828	0.5120099	0.656	0.4841897	0.824	0.417	NA	10
Ciita1	0.828	0.5506171	0.656	0.5228593	0.75	0.284	NA	14
Amica11	0.828	0.5166991	0.656	0.4799825	0.75	0.336	NA	14
S100a1	0.827	0.5990936	0.654	0.5699577	0.97	0.576	NA	0
Gm17056	0.827	0.6189793	0.654	0.470173	0.818	0.466	NA	0
Itpr1	0.827	0.6021404	0.654	0.5406999	0.879	0.471	NA	3
Gm11690	0.827	0.3069378	0.654	0.2852822	0.678	0.22	NA	3
Pfkp	0.827	0.6826267	0.654	0.6575224	0.831	0.49	NA	5
Gzmb1	0.827	1.5152447	0.654	1.4215359	0.634	0.061	NA	8
Spc25	0.827	0.4158035	0.654	0.4300603	0.792	0.333	NA	10
Rora1	0.827	0.6520202	0.654	0.5439697	0.783	0.265	NA	15
Dusp53	0.827	0.5295481	0.654	0.4257844	0.957	0.497	NA	15
Emilin1	0.826	0.5409188	0.652	0.5728506	0.914	0.436	NA	0
Nt5e	0.826	0.3554107	0.652	0.3273283	0.79	0.308	NA	0
Pou2af1	0.826	0.793701	0.652	0.7803824	0.88	0.447	NA	2
Snn	0.826	0.7853834	0.652	0.73257	0.841	0.404	NA	2
Coro1a1	0.826	0.8538033	0.652	0.6738188	0.998	0.592	NA	3
Nr4a2	0.826	0.8615188	0.652	0.9005083	0.779	0.334	NA	5
Lck	0.826	1.4177414	0.652	1.2234146	0.775	0.248	NA	7
Tcf7	0.826	0.9463311	0.652	0.983018	0.727	0.069	NA	7
Cxcr42	0.826	1.118257	0.652	1.0481224	0.757	0.334	NA	8
Fgfr1	0.825	0.7380286	0.65	0.6725506	0.992	0.562	NA	0
Cd79a	0.825	1.0429094	0.65	1.1201335	0.994	0.527	NA	2
Plp2	0.825	0.5367274	0.65	0.481474	0.83	0.484	NA	3
4930523C07Rik2	0.825	0.9386483	0.65	1.0939497	0.94	0.428	NA	4
Gimap42	0.825	1.0742523	0.65	1.088755	0.86	0.514	NA	7
Ctsw	0.825	0.6221944	0.65	0.5798199	0.673	0.11	NA	7
Itgal	0.825	1.2990072	0.65	1.3109146	0.757	0.362	NA	8
Ptprcap4	0.825	1.1080477	0.65	1.1091216	0.876	0.473	NA	8
Gpr1321	0.825	0.6695962	0.65	0.6350131	0.844	0.492	NA	14
Gimap6	0.824	0.7437409	0.648	0.7982549	0.96	0.626	NA	2
Bcl11a1	0.824	0.689401	0.648	0.6708228	0.873	0.426	NA	3
Ptprcap2	0.824	0.9416912	0.648	1.0657824	0.902	0.455	NA	4
D16Ertd472e	0.824	1.0908262	0.648	1.0087601	0.673	0.323	NA	8
Asf1b	0.824	0.503294	0.648	0.5377687	0.792	0.241	NA	10
Fcgr3	0.823	0.7612858	0.646	0.7232669	0.999	0.552	NA	0
Gatm	0.823	0.7337355	0.646	0.6495722	0.866	0.496	NA	5
Tbc1d10c	0.822	0.5006952	0.644	0.416585	0.79	0.383	NA	3
Tpx2	0.822	0.5805315	0.644	0.6254003	0.774	0.247	NA	10
Ltc4s1	0.821	0.7809827	0.642	0.8328349	0.999	0.637	NA	1
Prkar2b	0.821	0.5016113	0.642	0.6282253	0.827	0.384	NA	3
Ets11	0.821	0.5937727	0.642	0.4682144	0.957	0.442	NA	3
Glipr2	0.821	0.5771472	0.642	0.5788597	0.768	0.339	NA	5
Ccdc34	0.821	0.5099316	0.642	0.5340356	0.836	0.326	NA	10
Ebf1	0.82	0.671536	0.64	0.9302864	0.991	0.495	NA	2

Nr4a3	0.82	0.8375534	0.64	0.8628608	0.753	0.431	NA	5
Itk	0.82	1.0961168	0.64	1.0064564	0.689	0.063	NA	7
Spc24	0.82	0.7657822	0.64	0.8315115	0.78	0.201	NA	10
Hjurp	0.82	0.3394924	0.64	0.3746131	0.855	0.542	NA	10
Shtn1	0.82	0.6788763	0.64	0.5882817	0.802	0.385	NA	11
Rfc41	0.82	0.556413	0.64	0.5608109	0.773	0.297	NA	12
Dnajc91	0.819	0.5903162	0.638	0.6553529	0.887	0.472	NA	12
Gsn2	0.819	0.5309373	0.638	0.5715776	0.781	0.263	NA	14
Plxdc2	0.818	0.7370413	0.636	0.7874831	0.996	0.572	NA	0
Man2b1	0.818	0.669683	0.636	0.6866589	0.997	0.694	NA	0
Dpep2	0.818	0.6838596	0.636	0.6149327	0.987	0.564	NA	0
Blk	0.818	0.6806298	0.636	0.6737556	0.817	0.474	NA	2
Coro1a2	0.818	0.8863771	0.636	0.9298772	0.981	0.619	NA	4
Cd83	0.818	0.765722	0.636	0.6655072	0.926	0.616	NA	11
Oxct1	0.818	0.6238309	0.636	0.5150296	0.86	0.525	NA	11
Ccnd2	0.817	0.642805	0.634	0.812168	0.939	0.585	NA	3
Gimap11	0.817	0.4997375	0.634	0.4431733	0.866	0.424	NA	3
2700094K13Rik	0.817	0.6003051	0.634	0.6815875	0.956	0.578	NA	10
Pttg1	0.817	0.5472878	0.634	0.5465183	0.679	0.156	NA	10
Cdc201	0.817	0.4986863	0.634	0.4819107	0.629	0.179	NA	12
Timp21	0.816	0.7270647	0.632	0.8348986	1	0.696	NA	1
Ikzf31	0.816	0.6312809	0.632	0.4659074	0.787	0.313	NA	3
Cd55	0.816	1.275858	0.632	1.3174077	0.863	0.517	NA	4
Daglb	0.816	0.5472268	0.632	0.564613	0.766	0.404	NA	5
H2afx	0.816	1.2617254	0.632	1.2413883	0.784	0.402	NA	12
Cdca7	0.816	0.4119908	0.632	0.4393209	0.732	0.203	NA	12
Cenpm1	0.816	0.4270561	0.632	0.4117583	0.67	0.223	NA	12
Ccl51	0.816	0.7330115	0.632	0.5104519	0.87	0.469	NA	15
Efnb2	0.815	0.5259273	0.63	0.507749	0.915	0.506	NA	0
Cplx2	0.815	0.4685526	0.63	0.4843083	0.706	0.158	NA	3
Prf1	0.815	1.3671553	0.63	1.2920914	0.639	0.033	NA	8
Ifitm2	0.814	0.7896892	0.628	0.7315673	1	0.641	NA	0
Il5ra1	0.814	0.3551167	0.628	0.3017886	0.713	0.201	NA	3
Cebpe	0.814	0.5446178	0.628	0.4938931	0.61	0.077	NA	5
Gatm1	0.814	0.6007146	0.628	0.5793303	0.969	0.509	NA	10
Dhrs3	0.813	0.6032312	0.626	0.5043867	0.965	0.513	NA	0
Tiam1	0.813	0.6982239	0.626	0.6508181	0.812	0.458	NA	5
Mis18bp1	0.813	0.4121458	0.626	0.3977232	0.73	0.326	NA	10
Tbc1d4	0.813	0.4885317	0.626	0.4396823	0.688	0.308	NA	14
Smpdl3a	0.812	0.7443189	0.624	0.7968205	1	0.697	NA	0
Comt	0.812	0.6060917	0.624	0.6097599	0.986	0.634	NA	0
Maf	0.812	0.531474	0.624	0.5243201	0.929	0.522	NA	1
Sapcd11	0.812	0.6609238	0.624	0.5725846	0.727	0.307	NA	4
Aspa	0.811	0.5701171	0.622	0.5240694	0.867	0.431	NA	0
Cd274	0.811	0.6768964	0.622	0.8183597	0.904	0.578	NA	3
Card111	0.811	0.4632821	0.622	0.3956424	0.766	0.31	NA	3
Smc4	0.811	0.8351469	0.622	0.8770913	0.937	0.619	NA	10
Tgfbi1	0.811	0.7393727	0.622	0.7031415	0.956	0.591	NA	10
Mbtd1	0.811	0.7703861	0.622	0.7018081	0.81	0.463	NA	11
Ltb4r12	0.811	0.6879106	0.622	0.801721	0.75	0.23	NA	14
Cd93	0.81	0.6001334	0.62	0.6464126	0.94	0.413	NA	0
Hp	0.81	0.4609005	0.62	0.4210195	0.855	0.388	NA	0
Zbtb32	0.81	0.6734959	0.62	0.6817629	0.792	0.384	NA	2
Bhlhe41	0.81	0.5358169	0.62	0.595378	0.845	0.409	NA	3
Adora2a	0.81	0.3456027	0.62	0.2509534	0.634	0.297	NA	3
H2-DMb22	0.81	0.9338231	0.62	0.9233981	0.931	0.475	NA	4
Furin	0.81	0.5984535	0.62	0.6369498	0.805	0.463	NA	5

Sema4a	0.81	0.5228369	0.62	0.6134266	0.748	0.336	NA	5
Ncapg	0.81	0.2835349	0.62	0.3073561	0.686	0.073	NA	10
Rnase61	0.81	0.8321806	0.62	0.836948	0.826	0.404	NA	11
Upb1	0.81	0.7293125	0.62	0.6951166	0.744	0.243	NA	11
Timd4	0.809	0.7288836	0.618	0.7700008	0.997	0.482	NA	0
H2-Aa	0.809	0.8815304	0.618	0.7670821	0.997	0.654	NA	2
Abhd17b	0.809	0.6415034	0.618	0.6242308	0.877	0.563	NA	2
Rhoh1	0.809	0.5010378	0.618	0.4173996	0.837	0.392	NA	3
A530032D15Rik	0.809	0.3802886	0.618	0.2881195	0.647	0.263	NA	3
Ralgps22	0.809	1.0429766	0.618	1.0232105	0.826	0.447	NA	4
Fam129a	0.809	0.5037067	0.618	0.5518334	0.727	0.23	NA	5
Sh2d1a	0.809	1.1676685	0.618	1.1513103	0.663	0.054	NA	8
Celf4	0.809	0.437432	0.618	0.444356	0.688	0.227	NA	14
Gm4951	0.808	0.4071792	0.616	0.2654819	0.763	0.284	NA	0
Cacna1e	0.808	0.5025652	0.616	0.4894828	0.802	0.436	NA	3
Ifng	0.808	0.6991297	0.616	0.7489339	0.67	0.045	NA	7
Serpinb6b	0.808	1.3197313	0.616	1.4646177	0.718	0.202	NA	8
Cd23	0.808	0.8988955	0.616	0.9859855	0.946	0.556	NA	8
E2f8	0.808	0.3656838	0.616	0.3538718	0.73	0.151	NA	10
Ptgis	0.807	0.7762084	0.614	0.9442872	0.967	0.477	NA	0
Osm	0.807	0.6882171	0.614	0.7241423	0.891	0.464	NA	0
Ralgps2	0.807	0.6596457	0.614	0.5770115	0.828	0.412	NA	2
Ext1	0.807	0.6425738	0.614	0.6965059	0.783	0.385	NA	5
Rab27b	0.807	0.7738002	0.614	0.832938	0.604	0.018	NA	8
Ezh22	0.807	0.7195862	0.614	0.6596356	0.763	0.403	NA	12
Slbp	0.807	0.6785731	0.614	0.6298127	0.835	0.403	NA	12
Ikzf22	0.807	0.6063841	0.614	0.4699593	0.652	0.172	NA	15
Pf4	0.806	0.9912621	0.612	0.9746314	0.992	0.593	NA	0
Tgfb2	0.806	0.7838618	0.612	0.9437981	0.997	0.545	NA	0
Glb1	0.806	0.52493	0.612	0.5332195	0.959	0.561	NA	0
Stap12	0.806	0.9969886	0.612	1.0355472	0.796	0.3	NA	4
Coro2a	0.806	0.4901167	0.612	0.4842528	0.635	0.28	NA	5
Atp11b1	0.806	1.2061944	0.612	1.2192932	0.777	0.456	NA	8
Tpx21	0.806	0.6714695	0.612	0.6449718	0.701	0.251	NA	12
Cdc451	0.806	0.3591381	0.612	0.3471901	0.639	0.258	NA	12
Il1a	0.805	0.6533795	0.61	0.5390307	0.927	0.614	NA	0
Ly6d1	0.805	0.9384612	0.61	0.9251574	0.787	0.282	NA	4
Gfra21	0.805	0.3987897	0.61	0.3932305	0.719	0.334	NA	11
2810417H13Rik1	0.805	1.6036376	0.61	1.553615	0.68	0.172	NA	12
Tubb4b	0.805	0.9278336	0.61	1.0402738	0.928	0.603	NA	12
Lmo41	0.804	0.5233004	0.608	0.6553859	0.802	0.471	NA	11
Cd209c	0.804	0.3186496	0.608	0.3238139	0.661	0.084	NA	11
Il1r2	0.804	0.4922916	0.608	0.4809048	0.656	0.185	NA	14
Evi5	0.803	0.6708669	0.606	0.6193858	0.943	0.567	NA	0
Gimap61	0.803	0.6218473	0.606	0.5852215	0.975	0.631	NA	3
Pik3ip1	0.803	0.6137825	0.606	0.473008	0.713	0.239	NA	3
Plcb1	0.803	0.6348878	0.606	0.7317616	0.856	0.508	NA	5
Ikzf4	0.803	0.3225178	0.606	0.2713447	0.622	0.022	NA	7
Adrbk2	0.803	0.6137127	0.606	0.634511	0.678	0.108	NA	11
Cenpw1	0.803	0.63774	0.606	0.6232823	0.722	0.271	NA	12
Creg1	0.802	0.6312712	0.604	0.6361972	0.997	0.689	NA	0
Smagp	0.802	0.4400171	0.604	0.387814	0.871	0.522	NA	1
Dusp10	0.802	0.4327781	0.604	0.4168214	0.808	0.484	NA	3
Sell2	0.802	1.1317905	0.604	1.0411996	0.838	0.511	NA	4
Bri3bp1	0.802	0.5625256	0.604	0.523288	0.818	0.455	NA	11
Cd2261	0.802	0.3103406	0.604	0.3688839	0.719	0.177	NA	11
Slfn5	0.802	0.5161824	0.604	0.5123853	0.812	0.431	NA	14

의생명과학부/박*환/2023-10-20 18:19:24

Adgre1	0.801	0.7487894	0.602	0.920464	0.999	0.579	NA	0
1810011O10Rik	0.801	0.7919493	0.602	0.8250389	0.957	0.603	NA	0
Ets1	0.801	0.5011946	0.602	0.5719705	0.919	0.436	NA	2
Bcl11a	0.801	0.6449225	0.602	0.5116252	0.741	0.438	NA	2
B3gnt5	0.801	0.5525916	0.602	0.4808529	0.744	0.342	NA	3
Rfc4	0.801	0.3540523	0.602	0.3398558	0.742	0.295	NA	10
Fcrls1	0.801	0.816772	0.602	0.9240374	0.719	0.306	NA	11
Rab11fip11	0.801	0.53222	0.602	0.6211959	0.769	0.41	NA	11
Cdca81	0.801	0.6994715	0.602	0.6971675	0.67	0.268	NA	12
Ncapd2	0.801	0.4496533	0.602	0.4621258	0.701	0.289	NA	12
Coro1a3	0.8	0.701394	0.6	0.4050499	0.982	0.62	NA	5
H2afv	0.8	0.7928287	0.6	0.8136333	0.937	0.619	NA	10
Igsf61	0.8	0.6649554	0.6	0.6492339	0.938	0.579	NA	14
Rab11fip12	0.8	0.6036799	0.6	0.5906737	0.781	0.413	NA	14
Ier3	0.799	0.675221	0.598	1.015074	0.993	0.649	NA	0
Pmp22	0.799	0.6622127	0.598	0.7140836	0.992	0.539	NA	0
Fam46a	0.799	0.6674619	0.598	0.6222858	0.995	0.599	NA	0
Fam20c	0.799	0.5758532	0.598	0.5635934	0.959	0.483	NA	0
Metrnl	0.799	0.6912817	0.598	0.5319856	0.981	0.613	NA	0
Gimap3	0.799	0.5110384	0.598	0.6490093	0.872	0.474	NA	2
Mrc11	0.799	1.1415463	0.598	1.1770061	0.81	0.462	NA	11
Pak1	0.799	0.7991827	0.598	0.7939775	0.711	0.345	NA	11
Amica1	0.799	0.7637893	0.598	0.718919	0.727	0.333	NA	11
Msr1	0.798	0.6657462	0.596	0.6754938	0.997	0.572	NA	0
Hk2	0.798	0.5872878	0.596	0.4920432	0.907	0.575	NA	0
Pilrb2	0.798	0.4971928	0.596	0.4712399	0.904	0.416	NA	0
Cdkn1a	0.798	0.6866955	0.596	0.4346906	0.961	0.64	NA	0
Nrp1	0.798	0.4571011	0.596	0.4144238	0.816	0.396	NA	1
Bcl2a1a1	0.798	0.8613804	0.596	0.8455791	0.752	0.337	NA	11
Kif231	0.798	0.5481684	0.596	0.5517136	0.598	0.105	NA	12
Phgdh2	0.797	0.4644511	0.594	0.6059657	0.907	0.318	NA	12
Rfc3	0.797	0.3922945	0.594	0.3579843	0.66	0.227	NA	12
Ccr3	0.797	2.6896713	0.594	2.6687813	0.667	0.214	NA	13
Txk2	0.797	0.4389659	0.594	0.4027617	0.826	0.393	NA	15
Lpl	0.796	0.693961	0.592	0.6023534	0.931	0.537	NA	0
Cd5l1	0.796	0.8491321	0.592	0.8019748	0.963	0.525	NA	1
Serpinb6a1	0.796	0.639678	0.592	0.5921768	0.971	0.595	NA	1
Blvrb1	0.796	0.5993664	0.592	0.5629544	0.983	0.647	NA	1
Rdh12	0.796	0.4003646	0.592	0.4763429	0.785	0.369	NA	3
Il12a	0.796	0.3852011	0.592	0.4219019	0.743	0.258	NA	3
Ikzf2	0.796	1.4871532	0.592	1.4232062	0.711	0.155	NA	7
Klra3	0.796	1.2880205	0.592	1.3332536	0.589	0.035	NA	8
Cdk1	0.796	0.8347393	0.592	0.8575492	0.78	0.268	NA	10
Hells	0.796	0.3752557	0.592	0.3987678	0.761	0.418	NA	10
Cks21	0.796	0.8761958	0.592	0.8315281	0.753	0.385	NA	12
Mcm41	0.796	0.4650491	0.592	0.4476196	0.742	0.346	NA	12
Hells1	0.796	0.4092859	0.592	0.3909961	0.753	0.42	NA	12
Ablim1	0.795	0.511168	0.59	0.518901	0.914	0.547	NA	3
Diras2	0.795	0.5717788	0.59	0.4868832	0.657	0.27	NA	3
Rora	0.795	1.5619828	0.59	1.4550725	0.692	0.252	NA	7
Dna2	0.795	0.4311352	0.59	0.418259	0.678	0.31	NA	11
Racgap11	0.795	0.6325776	0.59	0.5943843	0.701	0.318	NA	12
Stab1	0.794	0.4634616	0.588	0.4028259	0.84	0.45	NA	1
Cxcr5	0.794	0.4465233	0.588	0.478054	0.817	0.409	NA	3
Lsr	0.794	0.4817479	0.588	0.4267091	0.621	0.228	NA	5
Hfe	0.794	0.6986489	0.588	0.7107653	0.802	0.369	NA	11
Smc21	0.794	0.8681867	0.588	0.8270541	0.732	0.389	NA	12

Hmgn2l	0.794	0.6196259	0.588	0.6782438	0.856	0.409	NA	12
Tnfrsf18l	0.794	0.3495575	0.588	0.294404	0.609	0.127	NA	15
Igsf8	0.793	0.5082035	0.586	0.4534889	0.947	0.611	NA	0
Ninj1l	0.793	0.6482483	0.586	0.7406085	0.995	0.663	NA	1
Phgdh	0.793	0.5317843	0.586	0.5464135	0.704	0.262	NA	2
Ptp4a3	0.793	0.5876197	0.586	0.5538537	0.874	0.573	NA	3
Nr4a2l	0.793	0.8769861	0.586	0.8391029	0.785	0.353	NA	11
Cfh	0.792	0.637565	0.584	0.7534067	0.999	0.592	NA	0
Padi4	0.792	0.5630331	0.584	0.4341089	0.81	0.373	NA	0
Msr1l	0.792	0.5807811	0.584	0.6083121	0.996	0.639	NA	1
Sulf2	0.792	0.6168263	0.584	0.6425126	0.719	0.282	NA	14
C3ar1	0.791	0.5569953	0.582	0.5266794	0.982	0.561	NA	0
Fcgrtl	0.791	0.5690677	0.582	0.6810326	0.994	0.659	NA	1
Emp1l	0.791	0.6270469	0.582	0.657904	0.98	0.624	NA	1
Dhfr	0.791	0.4717005	0.582	0.4423946	0.723	0.319	NA	10
Gpr132	0.791	0.6378286	0.582	0.5861989	0.81	0.49	NA	11
Sirpb1c	0.791	0.3694354	0.582	0.3761281	0.628	0.16	NA	11
Clspnl	0.791	0.5282101	0.582	0.5300177	0.629	0.187	NA	12
Tppp3l	0.791	0.4783245	0.582	0.4479781	0.625	0.252	NA	14
Cd5l	0.79	0.6657417	0.58	0.5882163	0.949	0.449	NA	0
Mertk	0.79	0.2667106	0.58	0.2646494	0.845	0.421	NA	0
Pltpl	0.79	0.6384824	0.58	0.7497587	0.998	0.648	NA	1
Nfatc1	0.79	0.6728718	0.58	0.6259616	0.782	0.472	NA	2
Hmgn3	0.79	0.3307157	0.58	0.3391182	0.684	0.294	NA	3
Cxxc5	0.79	0.3015175	0.58	0.2816088	0.648	0.235	NA	3
Klri2	0.79	0.9774385	0.58	1.0253545	0.594	0.015	NA	8
Arl4c2	0.79	0.9519929	0.58	0.948207	0.713	0.374	NA	8
Eps8l	0.79	0.5809589	0.58	0.6179053	0.893	0.592	NA	11
Cep55l	0.79	0.4539833	0.58	0.4606995	0.608	0.057	NA	12
Fam129a2	0.79	0.5287246	0.58	0.5754158	0.688	0.257	NA	14
Alox5ap	0.789	0.7169068	0.578	0.7514761	1	0.657	NA	0
Mafb1	0.789	0.5661032	0.578	0.6342029	0.983	0.577	NA	1
Lmo4	0.789	0.5345854	0.578	0.5724638	0.792	0.457	NA	5
Klrg1	0.789	0.7546338	0.578	0.7304342	0.545	0.024	NA	8
Itgb7l	0.789	0.6964648	0.578	0.6677351	0.893	0.506	NA	11
Coro1a5	0.789	0.7554252	0.578	0.6303365	0.975	0.636	NA	11
Ltc4s	0.788	0.755737	0.576	0.783431	0.999	0.57	NA	0
Sox5	0.788	0.5028931	0.576	0.4800376	0.693	0.203	NA	3
Satb1	0.788	1.2343974	0.576	1.0486959	0.739	0.364	NA	4
Fam107b	0.788	0.9637098	0.576	0.9269808	0.844	0.54	NA	7
2810417H13Rik	0.788	1.4059198	0.576	1.3797327	0.73	0.168	NA	10
Tmem176b1	0.788	1.2384593	0.576	1.3284222	0.826	0.499	NA	11
Col4a2	0.788	0.2945411	0.576	0.3224415	0.587	0.01	NA	11
Itgal2	0.788	0.7751548	0.576	0.8345204	0.781	0.369	NA	14
Lck2	0.788	0.3050502	0.576	0.2990875	0.739	0.264	NA	15
Gm20186	0.787	0.5480599	0.574	0.5256821	0.834	0.487	NA	0
Gimap1	0.787	0.6415857	0.574	0.5575347	0.786	0.427	NA	2
Ass1	0.787	0.4595246	0.574	0.4577293	0.749	0.269	NA	3
Traf1	0.787	1.0191852	0.574	1.026806	0.819	0.516	NA	7
Klrd1	0.787	1.1952804	0.574	0.8944311	0.686	0.192	NA	7
Cenpm	0.787	0.4043551	0.574	0.4008445	0.679	0.22	NA	10
Ckap2l	0.787	0.3748595	0.574	0.3678666	0.598	0.21	NA	12
Plac83	0.787	1.0223328	0.574	1.1503013	0.969	0.6	NA	14
Tnfaip2	0.786	0.5211798	0.572	0.5009239	0.886	0.484	NA	0
Rab32	0.786	0.418692	0.572	0.4385145	0.957	0.571	NA	0
Calml4	0.786	0.4663945	0.572	0.3999204	0.871	0.403	NA	1
Tmpo2	0.786	0.4915759	0.572	0.4907181	0.906	0.579	NA	14

C1qb	0.785	0.7728102	0.57	0.8423228	1	0.647	NA	0
Plod3	0.785	0.4792774	0.57	0.5841196	0.974	0.636	NA	0
Fcrla	0.785	0.6674181	0.57	0.6029321	0.822	0.468	NA	2
Incenp	0.785	0.6116727	0.57	0.5923775	0.799	0.413	NA	10
Cysltr11	0.785	0.6962631	0.57	0.6960485	0.835	0.441	NA	11
Zbtb322	0.785	0.4683923	0.57	0.5334509	0.897	0.436	NA	12
Pola11	0.785	0.2777219	0.57	0.2893965	0.629	0.233	NA	12
Tmem176a1	0.785	1.1810566	0.57	1.1880282	0.781	0.461	NA	14
Clec4b12	0.785	0.6172967	0.57	0.6581099	0.75	0.366	NA	14
Pi16	0.784	0.3987357	0.568	0.4166899	0.844	0.458	NA	0
Fam20c1	0.784	0.4775139	0.568	0.4631951	0.97	0.556	NA	1
Gimap41	0.784	0.5192001	0.568	0.4615012	0.887	0.476	NA	3
Bcl2	0.784	1.3225486	0.568	1.4199867	0.794	0.471	NA	7
Cd247	0.784	0.989976	0.568	0.9404212	0.673	0.017	NA	7
Mad2l1	0.784	0.3254866	0.568	0.3387281	0.686	0.252	NA	10
Hist1h1b	0.784	1.2313486	0.568	1.1791868	0.649	0.286	NA	12
Klrk13	0.784	0.3565602	0.568	0.2567353	0.783	0.314	NA	15
Slpi	0.783	1.6477011	0.566	1.3515463	0.854	0.539	NA	0
Fam46c	0.783	0.490349	0.566	0.4623771	0.735	0.416	NA	3
Lmo71	0.783	0.360419	0.566	0.397188	0.689	0.265	NA	3
Arl4c1	0.783	0.832341	0.566	0.7987596	0.781	0.368	NA	7
Tyms	0.783	0.6449692	0.566	0.6611756	0.761	0.33	NA	10
Atp11b	0.782	1.0005377	0.564	0.9021357	0.863	0.449	NA	7
Ccnb11	0.782	0.4993275	0.564	0.5002027	0.577	0.089	NA	12
Rasa4	0.782	0.4873181	0.564	0.4338192	0.75	0.418	NA	14
Gngt2	0.781	0.6368995	0.562	0.6625223	0.987	0.647	NA	0
Cd302	0.781	0.4011278	0.562	0.3772237	0.936	0.472	NA	0
Arhgap18	0.781	0.6183881	0.562	0.5827334	0.736	0.348	NA	11
Ednrb	0.78	0.7457648	0.56	0.809514	1	0.571	NA	0
Cd681	0.78	0.599445	0.56	0.6337808	0.993	0.671	NA	1
Pmp221	0.78	0.5957012	0.56	0.5476716	0.992	0.61	NA	1
Hmox1	0.78	0.5423528	0.56	0.489803	0.945	0.582	NA	1
Gm43291	0.78	0.6957913	0.56	0.7113721	0.691	0.204	NA	4
Slc16a3	0.78	0.4097726	0.56	0.4470322	0.661	0.311	NA	5
Smad7	0.78	0.6910416	0.56	0.7216266	0.702	0.262	NA	7
Cxcr41	0.78	0.4323337	0.56	0.5632262	0.806	0.327	NA	7
Hacd41	0.78	0.5374698	0.56	0.5376597	0.987	0.656	NA	10
Ms4a13	0.78	0.7156441	0.56	0.9627744	0.979	0.606	NA	12
Shisa8	0.78	0.2708617	0.56	0.2743298	0.649	0.153	NA	12
Colec12	0.779	0.4180311	0.558	0.3312871	0.904	0.543	NA	0
Gm43603	0.779	0.5492521	0.558	0.6402728	0.851	0.415	NA	2
Fgl2	0.779	0.3859073	0.558	0.4643884	0.66	0.28	NA	3
Gusb2	0.779	0.5048745	0.558	0.4933576	0.987	0.663	NA	10
AF251705	0.779	0.6144169	0.558	0.611257	0.62	0.157	NA	11
Net1	0.779	0.3450636	0.558	0.2947216	0.653	0.302	NA	11
Hr1	0.779	0.2718688	0.558	0.2720626	0.62	0.094	NA	11
Lockd	0.779	0.7069782	0.558	0.6983041	0.619	0.163	NA	12
Cdca31	0.779	0.6544181	0.558	0.6344637	0.629	0.16	NA	12
Ptms1	0.779	0.6680586	0.558	0.7585558	0.875	0.559	NA	14
F13a1	0.779	0.6663419	0.558	0.7267268	0.844	0.431	NA	14
Irf7	0.779	0.5643293	0.558	0.6036616	0.812	0.488	NA	14
Khk	0.778	0.5219593	0.556	0.6187123	0.91	0.458	NA	0
Nlrp3	0.778	0.4023899	0.556	0.5331163	0.929	0.592	NA	0
Plscr1	0.778	0.4401982	0.556	0.5682639	0.858	0.521	NA	3
Ero1lb1	0.778	0.5167927	0.556	0.4480615	0.846	0.49	NA	3
Xcl1	0.778	0.3736463	0.556	0.296875	0.625	0.094	NA	7
Cryl11	0.778	0.5069644	0.556	0.4609403	0.962	0.59	NA	10

Cdk11	0.778	0.5518764	0.556	0.5358666	0.649	0.273	NA	12
H2-DMb12	0.778	0.8356449	0.556	0.8530429	0.812	0.373	NA	14
Glipr11	0.778	0.4597077	0.556	0.3849831	0.719	0.344	NA	14
Tcn2	0.777	0.612504	0.554	0.7481122	1	0.624	NA	0
Engase	0.777	0.3990446	0.554	0.4901826	0.941	0.537	NA	0
Pf41	0.777	0.7313566	0.554	0.8064696	0.986	0.657	NA	1
Rabgap1l	0.777	0.616701	0.554	0.6909608	0.866	0.559	NA	2
Card11	0.777	0.4399166	0.554	0.4206984	0.663	0.316	NA	2
Ms4a6b	0.777	0.363574	0.554	0.2818171	0.831	0.489	NA	3
Cd72	0.777	0.6223433	0.554	0.6672774	0.668	0.291	NA	4
Ptgs12	0.777	0.5431968	0.554	0.5530055	0.987	0.643	NA	10
Napsa2	0.777	0.7290443	0.554	0.6505694	0.926	0.605	NA	11
Glipr1	0.777	0.4327955	0.554	0.3897212	0.678	0.341	NA	11
Sdf2l1	0.776	0.5469375	0.552	0.5267007	0.972	0.641	NA	0
Itsn1	0.776	0.4873995	0.552	0.4657973	0.96	0.527	NA	0
Sp140	0.776	0.5158077	0.552	0.5355399	0.92	0.613	NA	3
Rabgap1l1	0.776	0.4357416	0.552	0.4271036	0.885	0.564	NA	3
Abca9	0.776	0.5170236	0.552	0.5223358	0.75	0.368	NA	5
Il18rap	0.776	0.5988699	0.552	0.3871663	0.635	0.244	NA	7
Ccna2	0.776	0.763063	0.552	0.7636596	0.61	0.042	NA	10
Ndnf1	0.776	0.3037143	0.552	0.2529779	0.595	0.166	NA	11
Plp21	0.776	0.4561568	0.552	0.5238847	0.897	0.522	NA	12
Cd3g1	0.776	0.6056433	0.552	0.4305582	0.652	0.255	NA	15
Ninj1	0.775	0.6051905	0.55	0.659895	0.995	0.601	NA	0
Ptpn22	0.775	0.4712444	0.55	0.538562	0.898	0.578	NA	3
Nfatc11	0.775	0.4016672	0.55	0.3392979	0.82	0.474	NA	3
Dut	0.775	0.5965956	0.55	0.618827	0.786	0.354	NA	10
Rab322	0.775	0.5147875	0.55	0.4646737	0.981	0.68	NA	10
Prc1	0.775	0.6541146	0.55	0.6187868	0.629	0.185	NA	12
Ung	0.775	0.3822067	0.55	0.3664975	0.649	0.274	NA	12
Fcgr1	0.775	0.753875	0.55	0.6838217	0.75	0.422	NA	14
Plin2	0.774	0.5827801	0.548	0.6487981	0.993	0.593	NA	0
Blvrb	0.774	0.5228812	0.548	0.5026317	0.975	0.588	NA	0
Idh2	0.774	0.4713086	0.548	0.4607041	0.962	0.608	NA	10
Cep55	0.774	0.249548	0.548	0.2604212	0.579	0.054	NA	10
Birc51	0.774	1.1017153	0.548	1.0586782	0.619	0.085	NA	12
Coro1a6	0.774	0.6016974	0.548	0.7469661	1	0.637	NA	12
Dnmt11	0.774	0.560252	0.548	0.512386	0.753	0.401	NA	12
C4b	0.773	0.7105156	0.546	0.8826172	1	0.682	NA	0
Pygl	0.773	0.4931068	0.546	0.5946934	0.947	0.445	NA	0
Cpq	0.773	0.3705109	0.546	0.3857269	0.829	0.455	NA	0
Trem2	0.773	0.6106376	0.546	0.4846898	0.782	0.402	NA	1
Alkbh1	0.773	0.5045514	0.546	0.420983	0.811	0.438	NA	3
Clec4a21	0.773	0.871382	0.546	0.8017259	0.75	0.384	NA	14
Ms4a8a	0.772	0.4231005	0.544	0.4117618	0.952	0.542	NA	0
Bcam	0.772	0.2932544	0.544	0.3033341	0.849	0.393	NA	0
AC125149.3	0.772	0.3407673	0.544	0.3174065	0.718	0.393	NA	3
Arl4c	0.772	0.5120354	0.544	0.4755813	0.696	0.362	NA	5
Selp1	0.772	0.5783498	0.544	0.7409196	0.948	0.639	NA	6
Socs1	0.772	0.7210365	0.544	0.6697808	0.686	0.321	NA	7
Pilra	0.771	0.5057657	0.542	0.4631702	0.874	0.544	NA	0
Rcn3	0.771	0.2065513	0.542	0.2662498	0.76	0.341	NA	1
Gm8369	0.771	0.5454301	0.542	0.513885	0.805	0.446	NA	2
Gata3	0.771	1.4217155	0.542	1.2764812	0.622	0.015	NA	7
Lat	0.771	1.0744406	0.542	1.0383813	0.66	0.022	NA	7
1700025G04Rik1	0.771	0.6376206	0.542	0.7621566	0.589	0.171	NA	8
Asf1b1	0.771	0.5313147	0.542	0.5460584	0.649	0.246	NA	12

Dtl	0.771	0.4505874	0.542	0.4629947	0.649	0.21	NA	12
Plxnd12	0.771	0.4471298	0.542	0.449548	0.688	0.283	NA	14
App	0.77	0.6389592	0.54	0.6545007	1	0.654	NA	0
Ptgs1	0.77	0.5105909	0.54	0.6082046	0.98	0.51	NA	0
Igsf6	0.77	0.3758843	0.54	0.346492	0.858	0.463	NA	0
Cd36	0.77	0.4966964	0.54	0.6052371	0.985	0.579	NA	1
Cd69	0.77	0.5135845	0.54	0.5082873	0.842	0.538	NA	3
Prkcq1	0.77	1.0552722	0.54	1.0205275	0.644	0.269	NA	8
Crtap1	0.77	0.4252891	0.54	0.3925529	0.937	0.604	NA	10
Gins2	0.77	0.3259115	0.54	0.3225055	0.619	0.261	NA	12
Btla	0.769	0.4677394	0.538	0.5973755	0.698	0.292	NA	4
St3gal6	0.769	0.9552447	0.538	0.9100963	0.658	0.344	NA	8
Dctpp1	0.769	0.4731497	0.538	0.4009997	0.818	0.479	NA	10
Hirip31	0.769	0.286151	0.538	0.3039779	0.67	0.336	NA	12
Timeless	0.769	0.293221	0.538	0.300516	0.629	0.256	NA	12
Lsr1	0.769	0.3980081	0.538	0.4144452	0.688	0.248	NA	14
Nr4a22	0.769	0.4366231	0.538	0.3923529	0.719	0.357	NA	14
Ms4a6d	0.768	0.3232717	0.536	0.2958797	0.867	0.449	NA	0
Baiap2	0.768	0.4066602	0.536	0.259035	0.788	0.47	NA	1
Ltb1	0.768	0.5753431	0.536	0.4231766	0.942	0.543	NA	3
Cxcr4	0.768	0.8683837	0.536	0.9670765	0.761	0.316	NA	4
Ciita	0.768	0.403874	0.536	0.3340661	0.656	0.262	NA	4
Tppp3	0.768	0.3979504	0.536	0.3608286	0.636	0.248	NA	11
Csf1r	0.767	0.4924622	0.534	0.5199984	0.996	0.592	NA	0
Nxpe5	0.767	0.3691553	0.534	0.3936986	0.887	0.465	NA	1
Hepacam2	0.767	0.465462	0.534	0.3912529	0.637	0.313	NA	5
Satb11	0.767	0.858952	0.534	0.9287589	0.749	0.374	NA	7
Hirip3	0.767	0.2863423	0.534	0.2787205	0.686	0.334	NA	10
Bbc3	0.767	0.361026	0.534	0.2876234	0.711	0.362	NA	11
Shcbp1	0.767	0.3764511	0.534	0.37533	0.474	0.053	NA	12
St3gal5	0.766	0.5238223	0.532	0.7068019	0.993	0.568	NA	0
Cd2	0.766	0.6150915	0.532	0.7593455	0.951	0.502	NA	2
Ptprcap3	0.766	0.7624113	0.532	0.9471121	0.895	0.468	NA	7
Gm83692	0.766	0.6625971	0.532	0.8167097	0.867	0.483	NA	7
Rad51	0.766	0.2590466	0.532	0.3026998	0.692	0.148	NA	10
Tgfbi	0.765	0.4492122	0.53	0.4328053	0.934	0.53	NA	1
Sap30	0.765	0.390211	0.53	0.3720628	0.908	0.57	NA	1
Tnfrsf13c1	0.765	0.4375084	0.53	0.353092	0.77	0.346	NA	3
Cd931	0.765	0.5685614	0.53	0.7451579	0.874	0.554	NA	6
Ncapg2	0.765	0.3743799	0.53	0.4186328	0.711	0.177	NA	10
Melk	0.765	0.2294976	0.53	0.301986	0.597	0.028	NA	10
Prim11	0.765	0.423419	0.53	0.4754508	0.639	0.226	NA	12
Cdca5	0.765	0.2567061	0.53	0.2516726	0.608	0.249	NA	12
Fabp7	0.764	0.4721191	0.528	0.4876288	0.912	0.525	NA	0
Col18a1	0.764	0.3250479	0.528	0.3051581	0.741	0.312	NA	0
Tcn21	0.764	0.5577535	0.528	0.6949929	0.998	0.683	NA	1
Ahnak2	0.764	0.1917428	0.528	0.3138666	0.605	0.13	NA	3
Cks2	0.764	0.6512593	0.528	0.6889747	0.78	0.382	NA	10
Cd361	0.764	0.4735987	0.528	0.5340989	0.981	0.641	NA	10
F102	0.764	0.5285655	0.528	0.4158418	0.957	0.592	NA	15
Cd3d1	0.764	0.4707174	0.528	0.4089332	0.565	0.125	NA	15
Phgdh1	0.763	0.3564041	0.526	0.3418262	0.752	0.266	NA	3
Cd221	0.763	0.8922952	0.526	0.7072297	0.773	0.433	NA	4
Epb41l1	0.763	0.4952386	0.526	0.5199188	0.716	0.36	NA	5
Cd68	0.762	0.498062	0.524	0.478737	0.98	0.616	NA	0
Lilrb4a	0.762	0.4865078	0.524	0.4280968	0.979	0.671	NA	0
Ctsl1	0.762	0.5649003	0.524	0.6227852	0.998	0.691	NA	1

Cln8	0.762	0.3825231	0.524	0.2513699	0.764	0.422	NA	1
Gimap31	0.762	0.4100464	0.524	0.4517028	0.917	0.477	NA	3
Ptprcap1	0.762	0.4375919	0.524	0.4379105	0.926	0.421	NA	3
Ms4a12	0.762	0.598198	0.524	0.6709342	0.954	0.588	NA	4
Lpar1	0.762	0.5533649	0.524	0.5412186	0.691	0.303	NA	5
Cd226	0.762	0.427198	0.524	0.5115661	0.646	0.156	NA	5
Scn1b	0.761	0.4641269	0.522	0.514685	0.866	0.389	NA	0
Cgnl1	0.761	0.4013941	0.522	0.4463083	0.884	0.441	NA	0
Ctsf	0.761	0.3192827	0.522	0.3109082	0.831	0.464	NA	0
Chst7	0.761	0.4272881	0.522	0.3775929	0.586	0.167	NA	3
Stap11	0.761	0.4539984	0.522	0.3113551	0.71	0.278	NA	3
Ccnd11	0.761	0.5876217	0.522	0.5707203	0.925	0.57	NA	10
Ccdc341	0.761	0.4462349	0.522	0.433301	0.68	0.331	NA	12
AW1120102	0.761	0.5160706	0.522	0.5681588	0.739	0.413	NA	15
Fcer1g	0.76	0.5597692	0.52	0.5573544	1	0.63	NA	0
Hacd4	0.76	0.3822631	0.52	0.5413755	0.96	0.536	NA	0
Igf1	0.76	0.2895743	0.52	0.3723948	0.893	0.465	NA	0
Ms4a4c	0.76	0.8584242	0.52	0.7843833	0.68	0.238	NA	4
Slamf6	0.76	0.7079783	0.52	0.7835195	0.799	0.452	NA	4
Lst1	0.76	0.6339459	0.52	0.5670844	0.621	0.269	NA	5
Cxcl16	0.76	0.5753757	0.52	0.6015775	0.752	0.394	NA	11
Spink2	0.76	0.1335274	0.52	0.3053339	0.57	0.146	NA	11
Nusap1	0.76	0.920809	0.52	0.9123254	0.598	0.129	NA	12
Hopx1	0.76	0.3929119	0.52	0.4990378	0.781	0.442	NA	14
C1qc	0.759	0.6762019	0.518	0.8052666	1	0.649	NA	0
Emilin2	0.759	0.6246604	0.518	0.7950991	1	0.606	NA	0
Fam213b	0.759	0.4523626	0.518	0.4624057	0.84	0.473	NA	1
Cdca8	0.759	0.6079434	0.518	0.6272673	0.692	0.265	NA	10
Cdc20	0.759	0.4968106	0.518	0.5073679	0.648	0.175	NA	10
Kif221	0.759	0.3624742	0.518	0.3602107	0.526	0.18	NA	12
Arl4c3	0.759	0.7511972	0.518	0.7221113	0.719	0.38	NA	14
P2ry61	0.759	0.6897131	0.518	0.6832828	0.688	0.18	NA	14
Gadd45a	0.758	0.4123647	0.516	0.5906675	0.896	0.509	NA	0
4930455G09Rik	0.758	0.4654425	0.516	0.5213085	0.916	0.456	NA	0
Bri3bp	0.758	0.3622323	0.516	0.4051873	0.796	0.414	NA	3
Dennd5b	0.758	0.3186796	0.516	0.3046207	0.719	0.294	NA	3
Fcrla2	0.758	0.7910092	0.516	0.902311	0.83	0.497	NA	4
Myof	0.758	0.57195	0.516	0.6105019	0.818	0.507	NA	5
Camk4	0.758	0.889153	0.516	0.8176883	0.648	0.023	NA	7
Osbpl3	0.758	0.5286929	0.516	0.4237579	0.546	0.114	NA	7
Ikzf21	0.758	1.4599254	0.516	1.6241045	0.561	0.171	NA	13
Anxa3	0.757	0.4259668	0.514	0.481734	0.948	0.523	NA	0
Cflar	0.757	0.4793027	0.514	0.3365362	0.961	0.655	NA	0
Ednrb1	0.757	0.5751971	0.514	0.7354775	0.999	0.638	NA	1
Icam21	0.757	0.5720114	0.514	0.5780532	0.99	0.683	NA	1
Spib	0.757	0.6020568	0.514	0.5605957	0.785	0.392	NA	2
Itga63	0.757	0.6202788	0.514	0.5474529	1	0.653	NA	10
Tyms1	0.757	0.53101	0.514	0.5494845	0.649	0.334	NA	12
Nkg72	0.757	1.0976026	0.514	0.7055579	0.565	0.166	NA	15
Mafb	0.756	0.5957322	0.512	0.660956	0.972	0.507	NA	0
Lgals3bp	0.756	0.4863194	0.512	0.4913998	0.931	0.56	NA	0
Ifnar2	0.756	0.3990957	0.512	0.4538563	0.961	0.567	NA	0
Serpina3g	0.756	0.3107888	0.512	0.3101262	0.566	0.231	NA	2
Tnfrsf13c2	0.756	0.906116	0.512	1.0468406	0.787	0.372	NA	4
Ctla4	0.756	1.0887474	0.512	1.2743011	0.654	0.091	NA	7
Napsa3	0.756	0.5725753	0.512	0.8188538	0.969	0.605	NA	12
11-Sep	0.756	0.4347745	0.512	0.4470562	0.928	0.542	NA	12

Clec4e	0.755	0.5664922	0.51	0.5389769	0.965	0.645	NA	0
Gusb	0.755	0.4347022	0.51	0.4432955	0.961	0.545	NA	0
Sult1a1	0.755	0.3309007	0.51	0.3529452	0.753	0.315	NA	0
Csf1r1	0.755	0.5071242	0.51	0.5705852	0.999	0.655	NA	1
Rhoc	0.755	0.3614882	0.51	0.3323141	0.843	0.439	NA	1
Mertk1	0.755	0.2876352	0.51	0.3272703	0.894	0.478	NA	1
Gm15228	0.755	0.4098866	0.51	0.4847597	0.746	0.228	NA	3
Sell1	0.755	0.3138485	0.51	0.2651207	0.847	0.487	NA	3
Cx3cr1	0.755	0.5473971	0.51	0.488357	0.54	0.163	NA	5
Kif22	0.755	0.3491375	0.51	0.3734434	0.604	0.176	NA	10
Anpep	0.755	0.2835562	0.51	0.3728852	0.603	0.216	NA	11
Pros1	0.754	0.3432053	0.508	0.4292856	0.946	0.555	NA	0
Arg11	0.754	0.7695519	0.508	0.7053786	0.895	0.538	NA	1
Gsn	0.754	0.5171606	0.508	0.5126503	0.643	0.242	NA	5
Bcl11b	0.754	1.190134	0.508	1.1480681	0.689	0.055	NA	7
Fgl21	0.754	0.6172134	0.508	0.8912251	0.624	0.319	NA	8
P2ry10	0.754	0.2428935	0.508	0.3877256	0.818	0.445	NA	11
Kif111	0.754	0.5347891	0.508	0.5157094	0.619	0.176	NA	12
Vcan	0.754	0.4930165	0.508	0.4735185	0.625	0.263	NA	14
Itsn11	0.753	0.4007673	0.506	0.4768982	0.983	0.59	NA	1
Lgals3bp1	0.753	0.4514584	0.506	0.4317128	0.955	0.613	NA	1
Stap1	0.753	0.5582811	0.506	0.4032978	0.651	0.278	NA	2
Itgb7	0.753	0.4584841	0.506	0.3322401	0.802	0.472	NA	3
Ccr21	0.753	0.7936282	0.506	0.5720589	0.708	0.319	NA	7
Fam198b	0.753	0.2398377	0.506	0.2507047	0.786	0.378	NA	10
Plk2	0.752	0.6342294	0.504	0.7503004	0.987	0.668	NA	0
Adam151	0.752	0.4198825	0.504	0.4429013	0.981	0.677	NA	1
Pecam1	0.752	0.4788391	0.504	0.4273169	0.75	0.435	NA	3
Rnase6	0.752	0.4556645	0.504	0.4161504	0.769	0.361	NA	3
Ezh21	0.752	0.4255755	0.504	0.457494	0.83	0.4	NA	10
Colec122	0.752	0.3435456	0.504	0.3069392	0.95	0.645	NA	10
Cit	0.752	0.4798706	0.504	0.4789251	0.619	0.247	NA	12
Ndc80	0.752	0.4135278	0.504	0.422796	0.619	0.188	NA	12
Coro2a1	0.752	0.2864124	0.504	0.3036419	0.656	0.298	NA	14
Lgmn	0.751	0.3831048	0.502	0.6359642	0.995	0.557	NA	0
Klf9	0.751	0.4567993	0.502	0.4391044	0.916	0.582	NA	0
Cald1	0.751	0.3583299	0.502	0.3294867	0.886	0.422	NA	1
Lmo7	0.751	0.526574	0.502	0.5787536	0.636	0.264	NA	2
Foxm1	0.751	0.2896633	0.502	0.2868868	0.66	0.207	NA	10
Fyn1	0.751	0.480954	0.502	0.4336653	0.744	0.427	NA	11
Itgal1	0.751	0.4029379	0.502	0.3384396	0.702	0.366	NA	11
Blk2	0.751	0.428518	0.502	0.4983155	0.938	0.517	NA	12
Gusb1	0.75	0.3619315	0.5	0.3915379	0.968	0.609	NA	1
Fcrl1	0.75	0.269583	0.5	0.3681941	0.618	0.307	NA	2
Klrk12	0.75	0.5483662	0.5	0.4045842	0.636	0.311	NA	11
Glipr21	0.75	0.3410645	0.5	0.3666001	0.719	0.358	NA	11
Gda	0.749	0.4970127	0.498	0.5129051	0.998	0.646	NA	0
Cebpa	0.749	0.5223086	0.498	0.3589015	0.76	0.395	NA	0
Card191	0.749	0.4304486	0.498	0.4552421	0.977	0.675	NA	1
Parp1	0.749	0.4534701	0.498	0.4031494	0.795	0.481	NA	3
H2-DMb23	0.749	0.3192702	0.498	0.3629693	0.91	0.479	NA	5
Fyn	0.749	1.3095783	0.498	1.3828521	0.743	0.424	NA	8
Rad51ap1	0.749	0.2965587	0.498	0.3216281	0.648	0.078	NA	10
Avpi1	0.749	0.5935657	0.498	0.7099156	0.76	0.393	NA	11
Bhlhe411	0.749	0.31633	0.498	0.4014913	0.856	0.457	NA	12
Plin21	0.748	0.5007867	0.496	0.5900179	0.994	0.655	NA	1
Klrk1	0.748	0.662049	0.496	0.589127	0.654	0.304	NA	7

Lmo1	0.748	0.5762187	0.496	0.6260507	0.661	0.145	NA	11
Pmf12	0.748	0.3237685	0.496	0.3914106	0.897	0.43	NA	12
Fbxo5	0.748	0.3115719	0.496	0.3174659	0.515	0.192	NA	12
Pim3	0.748	0.7417583	0.496	0.7138873	0.844	0.503	NA	14
Igf11	0.747	0.4398823	0.494	0.5889301	0.933	0.525	NA	1
Cd281	0.747	1.0832056	0.494	1.071147	0.594	0.206	NA	8
Timd43	0.747	0.5475899	0.494	0.4538623	0.994	0.628	NA	10
Sgol1	0.747	0.2366306	0.494	0.2571554	0.585	0.067	NA	10
Mxd3	0.747	0.3628471	0.494	0.3571138	0.495	0.009	NA	12
Arl5c1	0.747	0.5662691	0.494	0.6211685	0.688	0.348	NA	14
Cryl1	0.746	0.4147844	0.492	0.4643308	0.898	0.469	NA	0
Ero1lb	0.746	0.5601991	0.492	0.5643506	0.805	0.489	NA	2
Pik3c2b	0.746	0.3283167	0.492	0.3241271	0.654	0.269	NA	3
Hr	0.746	0.4434358	0.492	0.4613329	0.573	0.073	NA	5
Diaph3	0.746	0.3222365	0.492	0.3528425	0.642	0.277	NA	10
Csrp1	0.746	0.3258785	0.492	0.2784998	0.588	0.273	NA	12
Slc11a11	0.745	0.399534	0.49	0.4961427	0.94	0.556	NA	1
Bcam1	0.745	0.3686749	0.49	0.3219236	0.873	0.46	NA	1
Serpina3g1	0.745	0.1789018	0.49	0.2986057	0.648	0.227	NA	3
Kazald1	0.745	0.5194839	0.49	0.4667097	0.523	0.146	NA	5
Prim1	0.745	0.2248969	0.49	0.2762011	0.654	0.223	NA	10
St3gal52	0.745	0.5228008	0.49	0.5450046	1	0.693	NA	15
Ifitm61	0.744	0.4103655	0.488	0.3022328	0.968	0.624	NA	1
Pmf1	0.744	0.5184634	0.488	0.5335567	0.736	0.386	NA	2
Birc5	0.744	1.226874	0.488	1.273767	0.635	0.081	NA	10
Tacc3	0.744	0.3733409	0.488	0.3721545	0.66	0.311	NA	10
Bub1	0.744	0.2504226	0.488	0.3102348	0.553	0.113	NA	10
Ccna21	0.744	0.6843712	0.488	0.6612349	0.515	0.047	NA	12
Gm26532	0.743	0.3340666	0.486	0.5610984	0.854	0.47	NA	3
Mcm6	0.743	0.5366163	0.486	0.5162271	0.692	0.324	NA	10
Clspn	0.743	0.3563752	0.486	0.3821129	0.642	0.184	NA	10
Rmi2	0.743	0.3596789	0.486	0.3466613	0.598	0.159	NA	12
Serpinb8	0.742	0.3150463	0.484	0.285113	0.774	0.38	NA	0
Rab11fip5	0.742	0.3739156	0.484	0.3569467	0.825	0.468	NA	1
Mdm1	0.742	0.2776514	0.484	0.2765714	0.833	0.522	NA	1
Timd42	0.742	0.5163141	0.484	0.5755635	0.935	0.619	NA	6
Dnajc9	0.742	0.2320586	0.484	0.3369767	0.893	0.469	NA	10
Atp8b4	0.742	0.2745892	0.484	0.2865057	0.653	0.278	NA	11
Camk1	0.741	0.3064136	0.482	0.3525765	0.856	0.465	NA	0
Lman1	0.741	0.3491842	0.482	0.3154988	0.892	0.554	NA	0
Emilin21	0.741	0.5495383	0.482	0.6066618	0.999	0.668	NA	1
Cd3021	0.741	0.4433359	0.482	0.4484519	0.961	0.54	NA	1
Gzmb	0.741	0.5771483	0.482	0.4553267	0.629	0.054	NA	7
Sh2d2a1	0.741	1.1951982	0.482	1.2417573	0.634	0.201	NA	8
Ifit2	0.741	0.4124156	0.482	0.3754596	0.562	0.207	NA	11
AI607873	0.74	0.3056507	0.48	0.3308915	0.882	0.509	NA	1
Sapcd1	0.74	0.4638287	0.48	0.379284	0.606	0.288	NA	2
Dapl1	0.74	0.2643612	0.48	0.3526715	0.565	0	NA	7
Gata31	0.74	0.5773508	0.48	0.7213062	0.515	0.025	NA	8
Phactr3	0.74	0.5524682	0.48	0.5384819	0.446	0.002	NA	8
Serpinb9b	0.74	0.4221828	0.48	0.5346758	0.455	0.006	NA	8
Aurkb	0.74	0.5047664	0.48	0.4743477	0.704	0.359	NA	10
Ppfibp1	0.74	0.410636	0.48	0.3789837	0.912	0.523	NA	10
Ncaph	0.74	0.2266489	0.48	0.2906214	0.635	0.183	NA	10
Ms4a4c1	0.74	0.8245116	0.48	0.790659	0.661	0.259	NA	11
Itsn12	0.74	0.3726884	0.48	0.3679338	0.957	0.654	NA	15
Fcna	0.739	0.4123715	0.478	0.4272095	0.995	0.597	NA	0

의생명과학부/박*환/2023-10-20 18:19:24

Gm16104	0.739	0.3450028	0.478	0.3277053	0.71	0.35	NA	0
Ikzf3	0.739	0.4916602	0.478	0.4526384	0.663	0.322	NA	2
Twsg1	0.739	0.3943917	0.478	0.4087256	0.709	0.311	NA	3
Slamf61	0.739	0.7170783	0.478	0.9038011	0.835	0.46	NA	7
Crtap	0.738	0.1923237	0.476	0.2587924	0.847	0.509	NA	0
Lyve1	0.738	0.4153457	0.476	0.4819263	0.694	0.285	NA	1
4930455G09Rik1	0.738	0.4443407	0.476	0.3890053	0.921	0.527	NA	1
Sbk1	0.738	0.424308	0.476	0.4413375	0.747	0.417	NA	3
Hepacam21	0.738	0.3749973	0.476	0.37076	0.711	0.326	NA	11
Prtn3	0.737	0.5161748	0.474	0.6402942	0.791	0.309	NA	0
Emp1	0.737	0.3830987	0.474	0.3755899	0.952	0.569	NA	0
Ifitm6	0.737	0.4719838	0.474	0.3672977	0.961	0.563	NA	0
Ptpn14	0.737	0.3203687	0.474	0.2570736	0.567	0.093	NA	4
Ezh2	0.737	0.4208089	0.474	0.3890252	0.698	0.397	NA	7
Nfic	0.737	0.4014037	0.474	0.3500342	0.912	0.571	NA	10
Cbx5	0.737	0.3082405	0.474	0.2920974	0.748	0.361	NA	10
Sell	0.736	0.2945057	0.472	0.4187176	0.811	0.485	NA	2
Sik1	0.736	0.3689469	0.472	0.5120402	0.816	0.413	NA	3
01-Sep	0.736	0.261272	0.472	0.4402802	0.846	0.505	NA	3
Cd3022	0.736	0.3646079	0.472	0.2634134	0.943	0.604	NA	10
Pttg11	0.736	0.4937549	0.472	0.4394572	0.495	0.162	NA	12
Casc5	0.736	0.3931006	0.472	0.3844362	0.546	0.119	NA	12
Batf32	0.736	0.7081601	0.472	0.6934786	0.625	0.217	NA	14
Pltp	0.735	0.4924271	0.47	0.3954039	0.999	0.583	NA	0
Fcgr31	0.735	0.5217049	0.47	0.5575643	0.998	0.623	NA	1
Themis	0.735	0.3797984	0.47	0.3664115	0.559	0.002	NA	7
Klrc1	0.735	0.520684	0.47	0.3164838	0.556	0.055	NA	7
Pcna	0.735	0.481995	0.47	0.4239101	0.736	0.381	NA	10
Clec5a	0.735	0.2475413	0.47	0.2699018	0.767	0.335	NA	10
C5ar1	0.734	0.388387	0.468	0.4994947	0.96	0.574	NA	0
Nupr11	0.734	0.4151561	0.468	0.5179776	0.984	0.555	NA	1
Plxnb2	0.734	0.3365129	0.468	0.3309523	0.969	0.647	NA	10
Dscc1	0.734	0.230621	0.468	0.2511534	0.526	0.097	NA	12
Osm1	0.734	0.5818727	0.468	0.5797741	0.957	0.589	NA	15
Fcer1g1	0.733	0.4588475	0.466	0.5329128	0.998	0.688	NA	1
Spib2	0.733	0.7029597	0.466	0.7017804	0.753	0.427	NA	4
Cd5l2	0.733	0.5046119	0.466	0.5575667	0.904	0.582	NA	6
Sh2d2a	0.733	0.6347813	0.466	0.65904	0.632	0.196	NA	7
Ifit21	0.733	0.415315	0.466	0.4201895	0.562	0.21	NA	14
Fcgrt	0.732	0.4463709	0.464	0.5219344	0.99	0.599	NA	0
Lgals3	0.732	0.4744584	0.464	0.3764635	0.991	0.659	NA	0
Dab2	0.732	0.4203042	0.464	0.4628409	0.98	0.659	NA	1
Fscn1	0.732	0.1310875	0.464	0.2536292	0.727	0.279	NA	1
Hmmr	0.732	0.5276511	0.464	0.5823422	0.528	0.033	NA	10
Pilrb21	0.732	0.3560876	0.464	0.2934315	0.931	0.554	NA	10
Pianp	0.732	0.3577845	0.464	0.3719685	0.727	0.398	NA	11
Spc241	0.732	0.6896186	0.464	0.6622267	0.567	0.207	NA	12
Fgd4	0.731	0.3338193	0.462	0.2893489	0.854	0.466	NA	0
Fpr2	0.731	0.2665314	0.462	0.2516169	0.727	0.314	NA	0
Lgmn2	0.731	0.558586	0.462	0.4868412	1	0.681	NA	10
Tmem141	0.73	0.3442765	0.46	0.3224011	0.829	0.521	NA	1
F101	0.73	0.4683025	0.46	0.4551405	0.962	0.587	NA	10
Mcemp11	0.73	0.404108	0.46	0.3530978	0.994	0.682	NA	10
Cdc45	0.73	0.2324025	0.46	0.2647412	0.66	0.255	NA	10
Fcgr4	0.73	0.4842106	0.46	0.4773039	0.688	0.383	NA	14
Serpinb101	0.73	0.2907243	0.46	0.32649	0.913	0.45	NA	15
Rab20	0.729	0.3941414	0.458	0.3646663	0.877	0.552	NA	0

Lrp11	0.729	0.4215769	0.458	0.4134897	0.973	0.642	NA	1
S100a11	0.729	0.3772562	0.458	0.3949159	0.97	0.638	NA	1
Lef1	0.729	0.798889	0.458	0.8724893	0.59	0.102	NA	7
Slc27a3	0.729	0.3644717	0.458	0.3300724	0.587	0.228	NA	11
Lrp1	0.728	0.3564547	0.456	0.4723793	0.964	0.584	NA	0
Ifitm21	0.728	0.5189959	0.456	0.5916753	0.999	0.698	NA	1
Fam46a1	0.728	0.4285266	0.456	0.5044353	0.996	0.661	NA	1
Cmtm3	0.728	0.3021884	0.456	0.2703205	0.829	0.506	NA	1
Nid1	0.728	0.4162939	0.456	0.505236	0.637	0.299	NA	2
Esco2	0.728	0.3805042	0.456	0.379405	0.516	0.061	NA	10
Raph1	0.728	0.2483769	0.456	0.2672263	0.83	0.461	NA	10
H2-DMb24	0.728	0.423218	0.456	0.4954092	0.901	0.497	NA	11
Ccl22	0.728	0.327969	0.456	0.3389417	0.504	0.008	NA	11
Ly6d2	0.728	0.5252413	0.456	0.672637	0.784	0.307	NA	12
Baiap21	0.728	0.3067826	0.456	0.2870032	0.87	0.522	NA	15
Colec121	0.727	0.3253746	0.454	0.323648	0.928	0.595	NA	1
Tnfrsf13c	0.727	0.545653	0.454	0.5689341	0.738	0.341	NA	2
Cd27	0.727	0.9212405	0.454	0.9325767	0.651	0.264	NA	7
Hist1h2ap	0.727	0.73658	0.454	0.6982288	0.485	0.144	NA	12
Esco21	0.727	0.4333488	0.454	0.4313116	0.485	0.065	NA	12
Rad511	0.727	0.3285463	0.454	0.3194021	0.567	0.152	NA	12
Ankrd33b	0.726	0.4232435	0.452	0.4431776	0.925	0.615	NA	0
Lgmn1	0.726	0.4313735	0.452	0.6206097	0.995	0.626	NA	1
Rhoh	0.726	0.4623117	0.452	0.4033019	0.71	0.403	NA	2
Slco3a1	0.726	0.3359924	0.452	0.4738067	0.565	0.043	NA	7
Kif11	0.726	0.5900112	0.452	0.5805582	0.654	0.173	NA	10
Axl	0.726	0.3088106	0.452	0.3141766	0.603	0.23	NA	11
Aldh1a21	0.726	0.2734522	0.452	0.2601838	0.57	0.176	NA	11
Plac82	0.726	0.5709536	0.452	0.9178303	1	0.597	NA	12
Klrd12	0.726	0.2516165	0.452	0.2749598	0.609	0.207	NA	15
Csf3r	0.725	0.306449	0.45	0.2559404	0.746	0.408	NA	0
Idh11	0.725	0.3118872	0.45	0.4440673	0.96	0.597	NA	1
Ms4a6d1	0.725	0.2704098	0.45	0.329337	0.905	0.507	NA	1
Foxp3	0.725	0.8629082	0.45	0.7703819	0.521	0.027	NA	7
Cenpf	0.725	0.8429687	0.45	0.9234622	0.616	0.191	NA	10
Ramp1	0.725	0.5559912	0.45	0.5153548	0.595	0.114	NA	11
Cd282	0.725	0.297653	0.45	0.3660019	0.565	0.213	NA	15
St3gal51	0.724	0.379549	0.448	0.4355177	0.989	0.636	NA	1
Pros12	0.724	0.3014182	0.448	0.3080573	0.975	0.665	NA	10
Ext11	0.724	0.4238967	0.448	0.4621098	0.711	0.403	NA	11
Tnfaip21	0.723	0.4026349	0.446	0.392447	0.918	0.541	NA	1
Pros11	0.723	0.2621519	0.446	0.3410052	0.956	0.614	NA	1
Gfra2	0.723	0.4832359	0.446	0.4871369	0.641	0.321	NA	5
Kif23	0.723	0.5046638	0.446	0.526099	0.566	0.102	NA	10
Arg12	0.723	0.5764283	0.446	0.4603589	0.906	0.592	NA	10
Cenpw	0.723	0.3477016	0.446	0.443162	0.711	0.268	NA	10
Ptgis1	0.723	0.485838	0.446	0.4060539	0.969	0.616	NA	10
Arg1	0.722	0.4939426	0.444	0.5744362	0.87	0.483	NA	0
Irf4	0.722	0.3484985	0.444	0.5147337	0.754	0.408	NA	2
Mzb12	0.722	0.4935351	0.444	0.6313171	0.871	0.459	NA	4
1700025G04Rik	0.722	0.4409742	0.444	0.407304	0.541	0.159	NA	5
Gimap12	0.722	0.6529269	0.444	0.7425882	0.797	0.466	NA	7
Cd932	0.722	0.4306953	0.444	0.4660121	0.957	0.568	NA	15
Xdh	0.721	0.2642597	0.442	0.2970541	0.885	0.528	NA	1
Ear2	0.721	0.5358844	0.442	0.7054138	0.552	0.086	NA	5
Ptgis2	0.721	0.3879674	0.442	0.39026	1	0.621	NA	15
Adam15	0.72	0.3352274	0.44	0.3484345	0.974	0.623	NA	0

Coro1a4	0.72	0.5714274	0.44	0.7010256	0.965	0.629	NA	7
Bcam2	0.72	0.3313977	0.44	0.2713556	0.843	0.523	NA	10
P2ry6	0.72	0.251403	0.44	0.2908977	0.603	0.176	NA	11
Tk1	0.72	0.2897523	0.44	0.3000387	0.495	0.129	NA	12
Rgl1	0.72	0.335143	0.44	0.2930716	0.719	0.357	NA	14
Ctla2a1	0.72	0.471513	0.44	0.4826438	0.652	0.257	NA	15
Bst11	0.719	0.2729316	0.438	0.3806005	0.949	0.641	NA	1
Fcmr2	0.719	0.4073176	0.438	0.5886313	0.887	0.462	NA	12
Mmp8	0.719	0.3028858	0.438	0.3017001	0.562	0.148	NA	14
Ccnd1	0.718	0.3196677	0.436	0.2785643	0.84	0.523	NA	1
Arhgap31	0.718	0.2145463	0.436	0.2558465	0.826	0.453	NA	1
Rab323	0.718	0.3171779	0.436	0.3052591	1	0.684	NA	15
Slc11a1	0.717	0.2811458	0.434	0.3533436	0.877	0.511	NA	0
Anxa31	0.717	0.378776	0.434	0.4630596	0.955	0.588	NA	1
Alox5	0.717	0.1765106	0.434	0.2994926	0.904	0.53	NA	1
Lgals3bp2	0.717	0.3211334	0.434	0.3396791	0.975	0.664	NA	10
Timd44	0.717	0.4609972	0.434	0.4653978	1	0.633	NA	15
Bst1	0.716	0.2845501	0.432	0.3593088	0.931	0.591	NA	0
Snx18	0.716	0.2758378	0.432	0.2751829	0.899	0.586	NA	0
Timd41	0.716	0.4576812	0.432	0.6856349	0.996	0.563	NA	1
Rab321	0.716	0.356515	0.432	0.3777429	0.973	0.628	NA	1
Akr1c18	0.716	0.4816061	0.432	0.4709833	0.471	0.078	NA	5
Sytl3	0.716	0.8421475	0.432	0.8135386	0.535	0.022	NA	8
Cxcl13	0.716	0.9649594	0.432	0.5980899	0.591	0.244	NA	10
Kif15	0.716	0.4018941	0.432	0.3995441	0.572	0.096	NA	10
Pilra1	0.716	0.3589606	0.432	0.3632087	0.943	0.637	NA	10
Kif20b1	0.716	0.3151704	0.432	0.334186	0.557	0.234	NA	12
Clec4d1	0.715	0.396071	0.43	0.5186079	0.993	0.658	NA	1
Il5ra	0.715	0.3480466	0.43	0.3317796	0.602	0.207	NA	2
Nid11	0.715	0.2744568	0.43	0.2863486	0.683	0.3	NA	3
Hist1h4d	0.715	0.3607999	0.43	0.4157286	0.667	0.313	NA	10
Mafb2	0.715	0.5331258	0.43	0.5217225	0.957	0.643	NA	15
Calml41	0.715	0.3096595	0.43	0.2711039	0.783	0.48	NA	15
Fyb	0.714	0.2845877	0.428	0.3398915	0.956	0.57	NA	0
Ltbp11	0.714	0.3747661	0.428	0.272242	0.937	0.622	NA	10
Cd209b	0.713	0.2309678	0.426	0.3083117	0.775	0.4	NA	0
Cfh1	0.713	0.4115821	0.426	0.5492787	0.995	0.657	NA	1
C3ar11	0.713	0.3583295	0.426	0.5376481	0.988	0.626	NA	1
Cmklr1	0.713	0.2821261	0.426	0.2884955	0.823	0.435	NA	1
Atp1a3	0.713	0.213921	0.426	0.2559158	0.835	0.46	NA	1
Sdc1	0.713	0.6664028	0.426	0.6156782	0.593	0.183	NA	5
Ube2c	0.713	1.3922324	0.426	1.4454174	0.642	0.203	NA	10
Kif20b	0.713	0.3906663	0.426	0.3401703	0.585	0.232	NA	10
Maf1	0.713	0.3380768	0.426	0.2752856	0.893	0.584	NA	10
Ccnd21	0.713	0.3931342	0.426	0.6413433	0.948	0.624	NA	12
Parp11	0.713	0.2457174	0.426	0.4230645	0.928	0.515	NA	12
Camk11	0.712	0.3189719	0.424	0.4436574	0.932	0.511	NA	1
Ifnar21	0.712	0.3199404	0.424	0.3628864	0.96	0.629	NA	1
Pola1	0.712	0.3173731	0.424	0.2883445	0.616	0.231	NA	10
Rnd3	0.712	0.4229127	0.424	0.4145968	0.752	0.436	NA	11
Kif151	0.712	0.3546657	0.424	0.3421902	0.515	0.099	NA	12
Ablim11	0.712	0.2574537	0.424	0.2882838	0.938	0.587	NA	12
Ldlr	0.712	0.5675785	0.424	0.5481815	0.781	0.473	NA	14
Selp2	0.712	0.4325686	0.424	0.4321964	0.957	0.653	NA	15
Card19	0.711	0.2329578	0.422	0.3830829	0.961	0.625	NA	0
Maged1	0.711	0.6480458	0.422	0.5349646	0.527	0.21	NA	5
A530064D06Rik	0.711	0.3707016	0.422	0.3528112	0.484	0.15	NA	5

Thy11	0.711	0.3287848	0.422	0.3210069	0.416	0.102	NA	8
Slc11a12	0.711	0.3238156	0.422	0.2818496	0.925	0.614	NA	10
Tlr13	0.71	0.2527424	0.42	0.3139404	0.809	0.363	NA	0
Dhrs31	0.71	0.3447564	0.42	0.4218561	0.977	0.582	NA	1
Serpinb10	0.71	0.3034213	0.42	0.3750357	0.835	0.375	NA	1
Epcam	0.71	0.2815653	0.42	0.4137259	0.586	0.166	NA	2
Gpr174	0.71	0.336306	0.42	0.3641036	0.657	0.158	NA	7
Cenpe	0.71	0.754905	0.42	0.7781109	0.597	0.25	NA	10
Tmem1411	0.71	0.3148328	0.42	0.2759102	0.899	0.566	NA	10
Gimap44	0.71	0.3423299	0.42	0.3917932	0.938	0.521	NA	12
Itga61	0.709	0.4806133	0.418	0.4331898	0.989	0.594	NA	1
Scn1b1	0.709	0.3474781	0.418	0.2520815	0.815	0.474	NA	1
Emilin11	0.709	0.3091323	0.418	0.2864774	0.918	0.571	NA	10
Emp2	0.709	0.492931	0.418	0.491725	0.368	0.007	NA	13
Engase1	0.709	0.2963751	0.418	0.3171917	1	0.656	NA	15
Neurl31	0.709	0.3408804	0.418	0.3160365	0.957	0.632	NA	15
Serpinb6a	0.708	0.3203489	0.416	0.3052543	0.918	0.547	NA	0
Kcnk13	0.708	0.1795586	0.416	0.2558019	0.73	0.308	NA	0
Gimap7	0.708	0.955072	0.416	1.0449482	0.673	0.17	NA	7
Plxdc22	0.708	0.4586589	0.416	0.3780826	0.994	0.692	NA	10
Hmmr1	0.708	0.5191804	0.416	0.5045575	0.464	0.037	NA	12
Lcp2	0.707	0.3219024	0.414	0.2912777	0.914	0.588	NA	0
C5ar11	0.707	0.3585739	0.414	0.3952188	0.968	0.633	NA	1
Ncaph1	0.707	0.3289414	0.414	0.3131142	0.505	0.187	NA	12
Sepp1	0.706	0.3113258	0.412	0.2945392	0.996	0.582	NA	0
Pygl1	0.706	0.3374739	0.412	0.3215667	0.926	0.528	NA	1
Prkcq	0.706	0.9638983	0.412	0.9831217	0.657	0.264	NA	7
Xcl11	0.706	1.5207605	0.412	1.6679405	0.584	0.101	NA	8
B3gnt51	0.706	0.1988962	0.412	0.2807242	0.742	0.386	NA	12
Rarres1	0.706	0.5009087	0.412	0.4967549	0.368	0.027	NA	13
Xcl12	0.706	0.4489503	0.412	0.5507819	0.478	0.111	NA	15
Adam8	0.705	0.1902891	0.41	0.279203	0.862	0.519	NA	0
Ptgs11	0.705	0.3533601	0.41	0.3823405	0.978	0.584	NA	1
Nav1	0.705	0.2772866	0.41	0.2905388	0.867	0.528	NA	1
Klrc2	0.705	0.6692095	0.41	0.5086834	0.457	0.019	NA	7
Klrb1a	0.705	0.8781141	0.41	0.8318244	0.48	0.075	NA	8
Lbp	0.705	0.258322	0.41	0.275541	0.616	0.307	NA	10
Snn2	0.705	0.3429002	0.41	0.5576176	0.897	0.46	NA	12
Lgmn3	0.705	0.4389565	0.41	0.4218556	1	0.686	NA	15
C5ar12	0.705	0.2849524	0.41	0.3270304	1	0.687	NA	15
Neurl3	0.704	0.4164438	0.408	0.4871709	0.88	0.529	NA	0
Idh1	0.704	0.2662226	0.408	0.3633561	0.928	0.543	NA	0
Plxdc21	0.704	0.3968453	0.408	0.4262367	0.986	0.64	NA	1
Bach2	0.704	0.9779101	0.408	0.9906122	0.62	0.257	NA	4
Bub1b	0.704	0.3604359	0.408	0.4049804	0.56	0.029	NA	10
Gimap13	0.704	0.3029324	0.408	0.3996296	0.866	0.473	NA	12
Gimap62	0.704	0.3089721	0.408	0.387459	0.979	0.669	NA	12
Spry21	0.704	0.1904582	0.408	0.5442925	0.696	0.356	NA	15
Naip1	0.703	0.2267562	0.406	0.3076933	0.788	0.42	NA	0
Flnb1	0.703	0.3240911	0.406	0.3854698	0.991	0.639	NA	1
Fabp72	0.703	0.4550129	0.406	0.5409876	0.937	0.634	NA	10
Adgre11	0.703	0.4841162	0.406	0.4560073	1	0.698	NA	10
Cxcr3	0.702	0.541425	0.404	0.7235516	0.594	0.066	NA	7
Cdca3	0.702	0.5550105	0.404	0.6048197	0.61	0.157	NA	10
Nrp11	0.702	0.3024542	0.404	0.3559717	0.843	0.459	NA	10
Cd300a	0.701	0.2800906	0.402	0.3388732	0.812	0.4	NA	0
Fabp71	0.701	0.2775603	0.402	0.3968898	0.913	0.585	NA	1

Ccnb1	0.701	0.4472763	0.402	0.4388575	0.516	0.087	NA	10
Hist1h1e	0.701	0.5020424	0.402	0.5111319	0.732	0.409	NA	12
Apoc1	0.701	0.4674178	0.402	0.709516	0.739	0.384	NA	15
Ptgs2	0.287	0.5780492	0.426	0.5552013	0.175	0.559	NA	13
Isg151	0.264	0.5727046	0.472	0.3424325	0.246	0.654	NA	13

Supplementary table S2. ScRNA seq analysis of total peritoneal cells.

LPM1

**Gene**	**p_val**	**avg_log2FC**	**pct.1**	**pct.2**	**p_val_adj**
Retnla	1.88E-251	1.001635	0.99	0.772	3.01E-247
Gdpd3	0	0.879515	0.844	0.203	0
Cd74	8.93E-263	0.84162	0.919	0.607	1.43E-258
Cd200r4	0	0.836693	0.873	0.207	0
H2-Aa	1.01E-236	0.757922	0.806	0.31	1.63E-232
H2-Ab1	1.52E-179	0.620155	0.754	0.334	2.44E-175
Fabp5	3.46E-147	0.601948	0.63	0.18	5.56E-143
Cd81	3.69E-209	0.598576	0.843	0.333	5.93E-205
Uba52	3.17E-282	0.531987	0.971	0.756	5.08E-278
Serping1	7.31E-92	0.513895	0.274	0.003	1.17E-87
AI467606	3.57E-192	0.511318	0.834	0.416	5.74E-188

LPM2

Gene	p_val	avg_log2FC	pct.1	pct.2	p_val_adj
Gm8797	3.78E-142	0.540434	0.747	0.097	6.06E-138
Uba52	4.59E-153	0.542337	0.969	0.704	7.38E-149
Igfbp4	2.02E-124	0.60734	0.918	0.533	3.24E-120
Fabp5	2.04E-108	0.611762	0.622	0.067	3.28E-104
Marco	1.18E-68	0.660639	0.489	0.079	1.90E-64
Retnla	3.37E-173	0.843935	0.999	0.926	5.41E-169
H2-Ab1	1.74E-133	0.856351	0.891	0.421	2.79E-129
Gdpd3	9.69E-183	0.902586	0.864	0.202	1.56E-178
H2-Aa	1.30E-156	0.957769	0.917	0.401	2.09E-152
Cd74	3.78E-158	0.987586	0.979	0.672	6.06E-154

SPM

Gene	p_val	avg_log2FC	pct.1	pct.2	p_val_adj
Uba52	2.42E-34	0.506196	0.946	0.592	3.89E-30
Prcp	1.61E-31	0.510331	0.961	0.57	2.58E-27
4930523C07Rik	2.94E-39	0.52544	0.705	0.116	4.71E-35
Calml4	2.76E-35	0.543855	0.535	0.063	4.42E-31
Epsti1	7.58E-45	0.546079	0.829	0.179	1.22E-40
Dnajc15	9.23E-38	0.550997	0.891	0.321	1.48E-33
A530064D06Rik	1.77E-29	0.555984	0.837	0.379	2.85E-25
Gm8797	6.02E-61	0.607799	0.783	0.085	9.67E-57
Cd300ld	9.70E-39	0.611803	0.938	0.423	1.56E-34
Ly6e	2.20E-26	0.61682	0.628	0.176	3.53E-22
Atp1lb	3.02E-38	0.631303	0.876	0.341	4.84E-34
Gbp2	1.56E-48	0.647946	0.767	0.123	2.50E-44
Clec10a	3.35E-48	0.676271	0.853	0.198	5.37E-44
AF251705	5.67E-49	0.704198	0.752	0.116	9.10E-45
9830107B12Rik	4.29E-90	0.983793	0.922	0.051	6.88E-86
Gdpd3	1.90E-74	1.064463	0.93	0.15	3.06E-70

ImM

Gene	p_val	avg_log2FC	pct.1	pct.2	p_val_adj
Gm8797	5.81E-40	0.519832	0.597	0.041	9.33E-36
Igfbp4	6.74E-15	0.546452	0.792	0.401	1.08E-10
Serping1	8.61E-30	0.548067	0.347	0.005	1.38E-25
H2-Ab1	1.57E-14	0.602019	0.708	0.336	2.52E-10
Marco	4.67E-25	0.641615	0.375	0.023	7.50E-21
Cd300ld	5.30E-27	0.670867	0.75	0.199	8.50E-23
H2-Aa	1.65E-22	0.75253	0.764	0.284	2.65E-18
Cd74	1.16E-18	0.78572	0.861	0.587	1.86E-14
Gdpd3	4.57E-40	0.920765	0.764	0.127	7.34E-36
Retnla	1.02E-27	1.052324	0.986	0.736	1.64E-23

Supplementary table S3. Upregulated DEGs in *Hdc*$^{+/+}$ mice relative to those in *Hdc*$^{-/-}$ mice in each cluster.

의생명과학부/박*환/2023-10-20 18:19:24
